# Goldfish phoenixin: (I) structural characterization, tissue distribution, and novel function as a feedforward signal for feeding-induced food intake in fish model

**DOI:** 10.3389/fendo.2025.1570716

**Published:** 2025-04-29

**Authors:** Xiangfeng Qin, Cheng Ye, Ying Wai Chan, Anderson O. L. Wong

**Affiliations:** School of Biological Sciences, The University of Hong Kong, Hong Kong, Hong Kong SAR, China

**Keywords:** phoenixin, feeding behaviors, body motility, appetite control, goldfish

## Abstract

Phoenixin (PNX) is a novel peptide with diverse functions mediated by the orphan receptor GPR173. It also plays a role in appetite control, but the effect is not consistent across species and the mechanisms involved are still unclear. Using goldfish as a model, the mechanisms underlying feeding regulation by PNX were examined. In our study, two isoforms of PNX, PNXa and PNXb, and one form of GPR173 were cloned in goldfish and found to be highly conserved compared to their counterparts in other species based on sequence alignment, phylogenetic analysis, and *in silico* protein modeling. Using RT-PCR, PNXa/b and GPR173 were confirmed to be ubiquitously expressed at the tissue level. In goldfish, transcript expression of PNXa/b and GPR173 in the liver and brain areas including the telencephalon, hypothalamus, and optic tectum, were elevated by food intake but suppressed by fasting. Intraperitoneal (IP) and intracerebroventricular (ICV) injections of PNX20a and PNX20b, the mature peptides for PNXa and PNXb respectively, were both effective in increasing foraging behavior, surface motility, and food intake. Furthermore, the expression of orexigenic factors (neuropeptide Y (NPY), agouti-related peptide, orexin, and apelin) was elevated with parallel drops in anorexigenic signals (cholecystokinin, pro-opiomelanocortin, corticotropin-releasing hormone, and melanin-concentrating hormone) in the telencephalon, hypothalamus, and/or optic tectum. In the same brain areas, receptor expression for anorexigenic factors (leptin and adiponectin) was attenuated with concurrent rises in receptor levels for orexigenic signals (NPY and ghrelin). In our study, after IP injection of PNX20a/b, downregulation of leptin, adiponectin, and other feeding inhibitors expressed in the liver was also noted. Our findings reveal that PNX20a/b can serve as an orexigenic factor in goldfish. PNX signals (both central and peripheral) can be induced by food intake and act within the brain to trigger foraging and food consumption via differential modulation of appetite-regulating factors and their receptors in different brain areas. The feeding responses observed may also involve a hepatic component with PNX repression of feeding inhibitors expressed in the liver. The PNX signals induced by feeding may form a feedforward loop to maintain/prolong food intake during a meal prior to the onset of satiation response in our fish model.

## 1 Introduction

Phoenixin (PNX) is a novel peptide first identified by bioinformatics (1) and produced as a cleavage product of its precursor small integral membrane protein 20 (SMIM20, also called MITRAC7) (2). In humans, SMIM20 is encoded by the C4orf52 gene located in chromosome 4 (3). The C-terminal cleavage of SMIM20 can lead to multiple forms of PNX, with PNX14 and PNX20 as the major products (4). Since a dibasic protein processing site (GRK) can be identified at the end of the PNX coding sequence (e.g., in mammals and avian species) (1), the C-terminus of PNX mature peptides is believed to be α amidated. C-terminal amidation appears to be crucial for the bioactivity of PNX, as the amidated PNX (but not its non-amidated form) is known to trigger biological functions in different assay systems, e.g., suppression of visceral pain in mouse model (5). PNX is widely expressed in the brain and peripheral tissues (6). In rats, a high level of PNX immunoreactivity can be detected in the hypothalamus and with notable signals in brain areas including the PVN, VMH, SON, ARC, and ME (1, 6). For peripheral tissues, the heart represents the organ with the highest level of PNX expression, although reduced levels of PNX immunoreactivity can still be noted in the intestine, stomach, pancreas, spleen, thymus, pituitary, and kidneys (1, 6). Recent studies have also confirmed PNX expression in the ovaries and periovarian adipose tissue, and a rise in ovarian PNX signal has been reported in rat model with polycystic ovary syndrome (7). The widespread distribution of PNX expression is consistent with the diverse functions documented for the peptide. In mammals, PNX is involved in a wide range of functions, including reproduction (1, 8), food intake (9, 10), drinking/thirst response (9, 11), insulin secretion (12), adipogenesis/adipocyte differentiation (13), memory retention (14), anxiety/anxiolytic action (15), stress response (16), immune functions (17, 18), and cardiac modulation/cardioprotection (19).

For the biological functions reported for PNX, the involvement of the orphan receptor GPR173 (also called SREB3) is well-documented. For examples, PNX-induced kisspeptin and GnRH expression in immortalized hypothalamic neurons (20) and PNX potentiation of LH secretion induced by GnRH in rat pituitary cells (8) can be negated by siRNA silencing of GPR173. GPR173 is a member of the SREB family of G protein-coupled receptors (GPCRs) and its protein sequence is highly conserved from fish to mammals (21). Similar to PNX, GPR173 is widely expressed at the tissue level, with high levels of expression in the olfactory areas of the forebrain, SON and PVN in the hypothalamus, and in peripheral tissues including the ovary and small intestine (22). Based on the study by Yosten’s group using a novel deductive ligand-receptor matching strategy followed by functional validation via GPR173 silencing in rat pituitary cells (23), the orphan receptor GPR173 has been proposed to be a putative receptor for PNX (8). The idea, however, is not supported by another study using comparative genomics and functional expression of GPR173 in HEK293 cells (24). Of note, the data available based on functional studies using the siRNA approach (8, 20) do support the idea that GPR173 is working downstream of PNX and mediates its biological functions. Although not much is known about the post-receptor signaling of GPR173, PNX has been reported to trigger biological actions via GPR173 coupled with the cAMP/PKA pathway and CREB phosphorylation, e.g., for PNX-induced GnRH expression in mHypoA-GnRH/GFP cells (20). Of note, a recent study in mice has shown that GPR173 can also act as the receptor for cholecystokinin (CCK) in mediating its enhancement on the inhibitory signals of GABAergic neurons in the auditory cortex (25), suggesting that GPR173 may be a “promiscuous receptor” with multiple ligands for its physiological functions.

Regarding the role of PNX in appetite control, a common consensus has not been reached as both stimulatory (10, 26) and inhibitory effects have been reported (27). In rats, feeding stimulation by PNX can be associated with the activation of nesfatin-1 neurons in brain areas including the SON, PVN, and NST (9). In contrast, PNX inhibits food intake in zebrafish, which is accompanied by a rise in hypothalamic cocaine- and amphetamine-regulated transcript (CART) with a parallel drop in ghrelin expression in the gut (27). Besides the PNX effect on feeding, species-specific variation in PNX expression caused by food deprivation has been reported in fish models (27, 28). In spotted scat, fasting induces PNX expression in the hypothalamus and this effect can be blocked by refeeding (28). Fasting in zebrafish, however, can reduce PNX expression in tissues including the brain, intestine, liver, gonads, and muscle (27). To date, the mechanisms underlying feeding regulation by PNX are not fully understood. In chickens, PNX can induce food intake and this hyperphagic effect is sensitive to the blockade of the neuropeptide Y (NPY) receptor in the brain and can be enhanced by central antagonism of the corticotropin-releasing hormone (CRH) receptor (26). Together with the findings in zebrafish with PNX-induced CART expression in the hypothalamus and ghrelin inhibition in the gut (27), we suspect that the differential effects of PNX on appetite control in different species are mediated by modifications of orexigenic/anorexigenic factors and their receptors expressed in different brain areas and peripheral tissues.

In this study, goldfish was used as a model to unveil the mechanisms underlying feeding regulation by PNX. Goldfish was selected for two reasons: (i) Most of the studies for PNX were based on mammals and there is a general lack of information for PNX functions in lower vertebrates including fish species, and (ii) goldfish is a well-documented lab model for cyprinids, the members of which (e.g., grass carp) are commercial fish with a high market value in Asian countries. As a first step, the structural identities of two forms of PNX, namely PNXa and PNXb, and one form of GPR173 [with sequence homology to GRP173a but not GPR173b reported in fish species (29, 30)] were established in goldfish by molecular cloning. After analyzing their structural features, tissue expression profile and phylogenetic relationship with their counterparts in other vertebrate classes, the transcript expression of PNXa/b and GPR173 in the liver and brain areas involved in feeding control was examined in goldfish with food intake or food deprivation to establish the link of PNX/GPR173 system with feeding status. After that, PNX20a and PNX20b, the mature peptides of PNXa and PNXb respectively, were synthesized and used to test their effects on feeding behaviors, food consumption, and body motility related to feeding after intraperitoneal (IP) or intracerebroventricular (ICV) injection in goldfish. To study the mechanisms for feeding control by PNX, IP and ICV injections of PNX20a/b were performed to investigate the effects of PNX on transcript expression of appetite-regulating factors and their receptors in the telencephalon, hypothalamus, and optic tectum. In the experiment with IP injection of PNX20a/b, parallel measurement of feeding regulators expressed in the liver was also conducted. Our studies not only reveal the neuro-endocrine components for appetite control by PNX but also shed light on a novel feedforward loop for feeding-induced food intake mediated by PNX, which may play a key role in maintaining or prolonging feeding during a meal prior to the onset of satiation response. Given that the feeding promotion by PNX is expected to have a beneficial effect on body growth, our findings will also have the potential for future applications in fish farming.

## 2 Materials and methods

### 2.1 Experimental animals

Goldfish (*Carassius auratus*) with a body weight of 30–45 g were acquired from local pet stores and maintained in our central aquarium for at least 4 weeks prior to any experimentation. During the period, the fish were housed in 200 L well-aerated water tanks at 20 °C under a 12-hr light:12-hr dark photoperiod and fed on a daily basis with fish feed provided in the form of floating pellets. Since the goldfish obtained were sexually regressed [gonadosomatic index (GSI) ≤ 0.25%] and with no apparent sexual dimorphism, fish of mixed sexes were used in our experiments. PNX treatment and tissue sampling were performed according to protocol CULATR 5495-20 approved by the Committee for Animal Use in Teaching and Research at the University of Hong Kong.

### 2.2 Molecular cloning, sequence analysis and tissue expression of goldfish PNX and GPR173

Goldfish PNXa (Accession No. XM_026268684), PNXb (Accession No. XM_026216912), and GPR173 (Accession No. XM_026270294) were cloned by 5’/3’ RACE using a GeneRacer kit (Thermo Fisher) with primers designed based on the conserved regions of zebrafish PNX and GPR173. Sequence alignment, 3D protein modeling, and phylogenetic analysis were conducted using Clustal W (http://www.ebi.ac.uk/clustalw), AlphaFold2 (https://colab.research.google.com/github/sokrypton/ColabFold/blob/1.2/alphaFold2.ipynb), and MEGA X (http://www.megasoftware.net/index.html), respectively. The snake plot for GPR173 was constructed using Protter software (https://wlab.ethz.ch/protter/start/). For intron/exon organization and comparative synteny of PNX genes, PNX and its neighboring genes in the same genomic scaffold of representative species were downloaded from the NCBI genome database and analyzed using Splicing Finder and Genomicus software (https://www.genomicus.bio.ens.psl.eu/genomicus-57.01/). For tissue expression of PNXa/b and GPR173, RT-PCR was performed in selected tissues and brain areas using primers and PCR conditions for PNXa, PNXb, and GPR173 as described in **Supplementary Table 1**. The authenticity of PCR products was also confirmed by Southern blot using DIG-labeled probes for the respective gene targets and RT-PCR for β actin was routinely conducted to serve as an internal control.

### 2.3 PNX and GPR173 expression associated with feeding and fasting in goldfish

To establish a functional link between the PNX/GPR173 system and feeding status in fish model, goldfish were individually housed in 25-liter water tanks and entrained for ≥ 14 days with a one-meal-per-day feeding regimen with food provision (~2.5% BW) at 10:00 AM (as time zero for our experiment). For our study to test the effect of food intake up to 6 hr on PNX and GPR173 expression at the tissue level, goldfish without provision of food pellets were used as the control while the fish with regular feeding (with food provision at 10:00 AM) were taken as the “feeding” group. In our reciprocal experiment, the effect of food deprivation was examined up to 10 days (as the “fasting” group) and the group with regular one-meal-per-day feeding was used as the control. At the time points indicated for the two experiments, liver samples and brain areas including the telencephalon, hypothalamus, and optic tectum were harvested. Total RNA was isolated using TRIzol (Invitrogen) followed by reverse transcription with SuperScript II (Thermo Fisher). RT samples prepared were then subjected to real-time PCR for PNXa, PNXb, and GPR173 expression using a QuantiTect SYBR Green RT-PCR kit (Qiagen) with a RotorGene Q qPCR System (Qiagen). PCR conditions and primers used for the respective gene targets (including QC data for product size, *T_m_* values, and PCR efficiency) were listed in **Supplementary Table 2**. In these studies, serial dilutions of plasmid DNA with the amplicon for the target gene were used as the standards and parallel real-time PCR for 18S RNA was used as the internal control.

### 2.4 PNX20a/b treatment on feeding behaviors, food intake, and body motility in goldfish

To test the effects of PNX on feeding control, PNX20a (AGINQADVQPAGVKIWSDPF-NH_2_) and PNX20b (AGVNQADVQPAGLKIWSDPF-NH_2_), the mature peptides of PNXa and PNXb respectively, were synthesized by Genscript (Piscataway, NJ) and used for whole animal experiments with peripheral administration via IP injection and central administration via ICV injection as described previously (31). In these studies, parallel injection of fish physiological saline was used as the control. For the experimental setup (**Supplementary Figure 1A**), goldfish singly housed in 25-liter water tanks and entrained with a one-meal-per-day feeding schedule were routinely provided with floating food pellets (~2% BW) at 10:00 AM (“time zero” for our experiments). After IP/ICV injection of PNX20a/b, the feeding behaviors caused by food provision were recorded with an AVD714 Network Video Surveillance System (AVTECH). In goldfish, three types of feeding behaviors can be recognized as previously described by Peter’s group (32), including complete feeding (surface foraging), incomplete feeding (food spitting), and bottom feeding (bottom foraging) (for details, please refer to (31)). Based on the videos captured, different types of feeding behaviors were scored for 2 hr in a single-blind manner. After the 2-hr period, the food pellets remaining were harvested and dried to constant mass in a 65°C oven. The difference in mass of the remaining pellets versus the pellets added at the beginning of the experiment was taken as the food consumption during the test period.

To investigate the effects of PNX on body motility associated with feeding, trajectory analysis was performed with the videos captured (15 frames/sec) for the preceding experiments with IP/ICV injection of PNX20a/b using the AI-assisted DeepLabCut ™ (http://www.mackenziemathislab.org/deeplabcut) (**Supplementary Figures 1B, C**). The software based on a convolutional neural network (CNN) model allows for supervised machine learning for pose estimation and tracking of positions in space and time (33). To train the CNN model for identification and tracking of body parts of interest, including the eyes, operculum, dorsal fin, pelvic fin, caudal fin and main torso, a total of six 4-minute video clips (three from the control and three from the group with IP injection of PNX20a) was used for model training with 0.5 × 10^6^ iterations. After refining the training dataset with repeated cycles of manual inspection and labeling missing body parts in representative frames with suboptimal threshold of likelihood, the final model was used to generate the coordinate datasets (X and Y coordinates for space and Z coordinates for time) for the respective body parts based on a 60-minute trajectory tracing from the videos captured for the feeding experiments with/without PNX20a/b treatment. Only the coordinate data for body parts with a likelihood threshold ≥ 0.9 were extracted for motility analysis and the missing data (i.e., the data with a likelihood threshold < 0.9, constituting only a small fraction of individual datasets) were imputed by linear interpolation based on the neighboring data points. The X and Y coordinates (in pixels) for different body parts were then used to calculate the “average coordinates” of the whole body in the same frame (i.e., the same time point in Z coordinates). With the average body coordinates as a marker for body movement, the data extracted for (i) the X-Y projection view along the Z-axis (for motility analysis of fish swimming) and (ii) the Y-Z projection view along the X-axis (for spatial preference of fish motility) were analyzed using the trajr toolkit in the R package (https://cran.r-project.org/web/packages/trajr/index.html) to calculate the total distance traveled, the average velocity at regular intervals, the duration of rapid movement, and the duration of fish movement in the upper/lower half of water body. Rapid movement was defined as the transient velocity over the tested period ≥ 3 SEM of the average velocity in the control group. For spatial preference analysis, the mid-level water depth (i.e., 50% from the water surface to the bottom) was used to divide the water body into the upper and lower halves. To visualize the data for motility analysis and spatial preference, trajectory plots for goldfish swimming were constructed using RinearnGraph3D (https://www.rinearn.com/en-us/graph3d/) and the heatmaps for clustering of motility activity were generated using ggplot2 in the R package (https://ggplot2.tidyverse.org).

### 2.5 Effect of PNX20a/b treatment on feeding regulators and their receptors expressed in different brain areas

To shed light on the mechanisms involved in feeding regulation by PNX, peripheral administration by IP injection and central administration by ICV injection of PNX20a and PNX20b were conducted 15 min prior to the scheduled feeding time (10:00 AM, but without food provision) in goldfish entrained with a one-meal-per-day feeding regimen. Parallel injection with fish physiological saline was used as the control. At the time points indicated, brain areas, including the telencephalon, hypothalamus, and optic tectum, were harvested for total RNA isolation, reverse transcription, and subsequent real-time PCR for transcript expression of appetite-regulating factors and their receptors. For the experiment with IP injection of PNX20a/b, liver samples were also collected at respective time points and subjected to the same procedures to monitor the transcript expression of feeding regulators at the hepatic level. Similar to the preceding section on the quantitation of PNX and GPR173 transcripts, real-time PCR for target genes was conducted with a QuntiTect SYBR Green RT-PCR kit (Qiagen) using a RotorGene Q qPCR System (Qiagen) with primers and PCR conditions as described in **Supplementary Table 2**. Serial dilutions of plasmid DNA with the amplicon for the target gene were used to construct the standard curve for calibration of transcript expression in our RT samples. After individual real-time PCR, melting curve analysis was routinely performed and the authenticity of PCR products was confirmed by the *Tm* value deduced. In our initial studies, transcript expression of 18S RNA, β actin, and GAPDH were also monitored. Since notable changes in β actin and GAPDH signals were observed after PNX treatment and 18S RNA expression in our samples was found to be quite stable, real-time PCR for 18S RNA was routinely used as the internal control for our experiments.

### 2.6 Data transformation and statistical analysis

For transcript expression of target genes, the raw data from real-time PCR (expressed as femtomole transcript detected/μl RT sample prepared by reversed transcription of 5 μg total RNA) were deduced from the respective standard curves (with dynamic range of 10^5^ and correlation coefficient of ≥0.95 using data calibration under unsupervised mode in RotorGene Q software v1.7 (Qiagen). The data were then normalized with 18S RNA expressed in the same sample and transformed as a percentage of the mean value in the time-matched control (as “%Ctrl”). The transformation was conducted to allow for the pooling of data from individual fish in the same group without increasing the overall variability of final results caused by variations in the basal level of target gene expression. Similar to the corresponding data for feeding behaviors, food intake, and body motility related to foraging, the data presented (mean ± SEM, N = 12) were analyzed with one-way ANOVA followed by the Newman–Keuls test (for dose-dependence studies) or two-way ANOVA followed by Bonferroni test (for time-course studies) using Prism 6.0 (GraphPad). Differences between groups were considered significant when *p* < 0.05.

## 3 Results

### 3.1 Structural characterization, 3D protein modeling, phylogenetic analysis, and tissue expression profiling of goldfish PNX and GPR173

**Figure 1:**
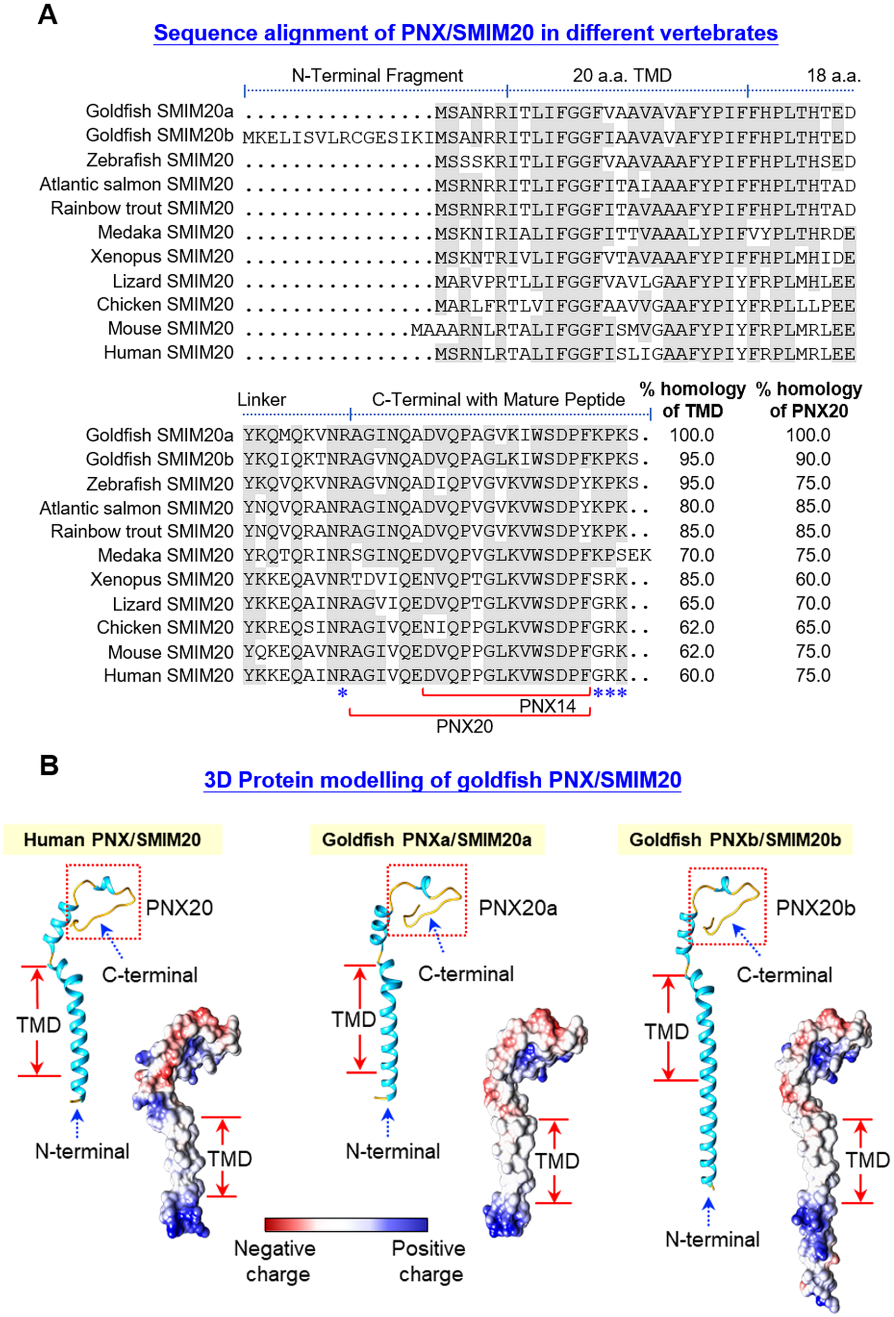
Sequence alignment and 3D protein modeling of goldfish PNX/SMIM20. **(A)** Sequence alignment of goldfish PNXa/SMIM20a and PNXb/SMIM20b with the corresponding sequences in other species. The a.a. sequences of SMIM20 in species from different vertebrate classes were downloaded from the NCBI genome database and aligned with those of goldfish PNXa/SMIM20a and PNXb/SMIM20b using the Clustal-W algorithm. Conserved a.a. residues were boxed in grey and the subdomain structures including the N-terminal, transmembrane domain (TMD), linker region, and the C-terminal with the mature peptide PNX20 and its truncated fragment PNX14 were delineated with the horizontal lines above the respective sequences. The monobasic/dibasic protein cleavage sites flanking the mature peptide PNX20 were marked by asterisks (*). **(B)** *In silico* protein modeling of goldfish PNXa/SMIM20a and PNXb/SMIM20b with their human counterpart as the reference. 3D Protein modeling of PNX/SMIM20 was constructed with AlphaFold and visualized using ChimeraX. The helical (light blue) and random coil structures (yellow) of the deduced models are shown in the ribbon plots and the charge distribution on the molecular surface (blue for positive charge, red for negative charge, and white for non-polar residues) is presented in the corresponding surface plots.

Using 5’/3’ RACE, two forms of PNX, PNXa/SMIM20a and PNXb/SMIM20b, and one form of GPR173 were cloned in goldfish. As shown in **Supplementary Figure 2**, PNXa cDNA (651 bp) is composed of an 84 bp 5’UTR, a 360 bp 3’UTR, and a 207 bp open reading frame (ORF) encoding a 68 a.a. PNXa precursor with a deduced MW of ~7.62 kDa. Similarly, PNXb cDNA (602 bp) is comprised of a 28 bp 5’UTR, a 319 bp 3’UTR, and a 255 bp ORF encoding an 84 a.a. PNXb precursor with a deduced MW of ~9.42 kDa. In these two cDNAs, multiple polyadenylation signals can be located in 3’URT (with actaaa and aataaa for PNXa; and gataaa, attaaa, and aataaa for PNXb). Based on the PNXa/b precursor proteins deduced from the respective ORFs, sequence alignment was performed with the a.a. sequences of SMIM20 reported in other species (**Figure 1A**). Structural analysis revealed that the PNX precursor SMIM20 could be divided into four domains, including a short N-terminal, a 20 a.a. transmembrane domain (TMD), an 18 a.a. linker, and the mature peptides (PNX20 and its truncated peptide PNX14) embedded in the C-terminal. Of note, the N-terminal in goldfish PNXb was found to be notably longer. Furthermore, the N-terminal sequences appeared to be quite variable across species and the same was also true for the 18 a.a. linker. In contrast, the regions covering TMD and mature peptides were shown to be highly conserved (with 60%–100% homology from fish to mammals). In the C-terminal, flanking of PNX20 sequences by mono/dibasic protein cleavage sites was consistently observed across different species. It would be logical to assume that PNX20a and PNX20b are the mature peptides of goldfish PNXa and PNXb, respectively. Based on the a.a. sequences of PNXa and PNXb, *in silico* protein modeling was conducted with AlphaFold (**Figure 1B**). Using human PNX as a reference, the 3D models of goldfish PNXa and PNXb were found to be highly comparable to their human counterparts. In both cases, the N-terminal and TMD regions formed an extended α helix linking with the mature peptide PNX20a/b via a short helix covering the linker region. For the region covering PNX20a/b (same for human PNX20), the mature peptide was shown to have a helical turn in the center with random coils sticking out at both ends. Except for the extended end in the N-terminal of PNX20b, the surface charge distribution of goldfish PNXa/b and human PNX, including (i) the hydrophobic surface of TMD, (ii) basic residues in the N-terminal, and (iii) the unique pattern of charge distribution in the mature peptide, was highly comparable (despite minor variations could still be noted in the linker region).

**Figure 2:**
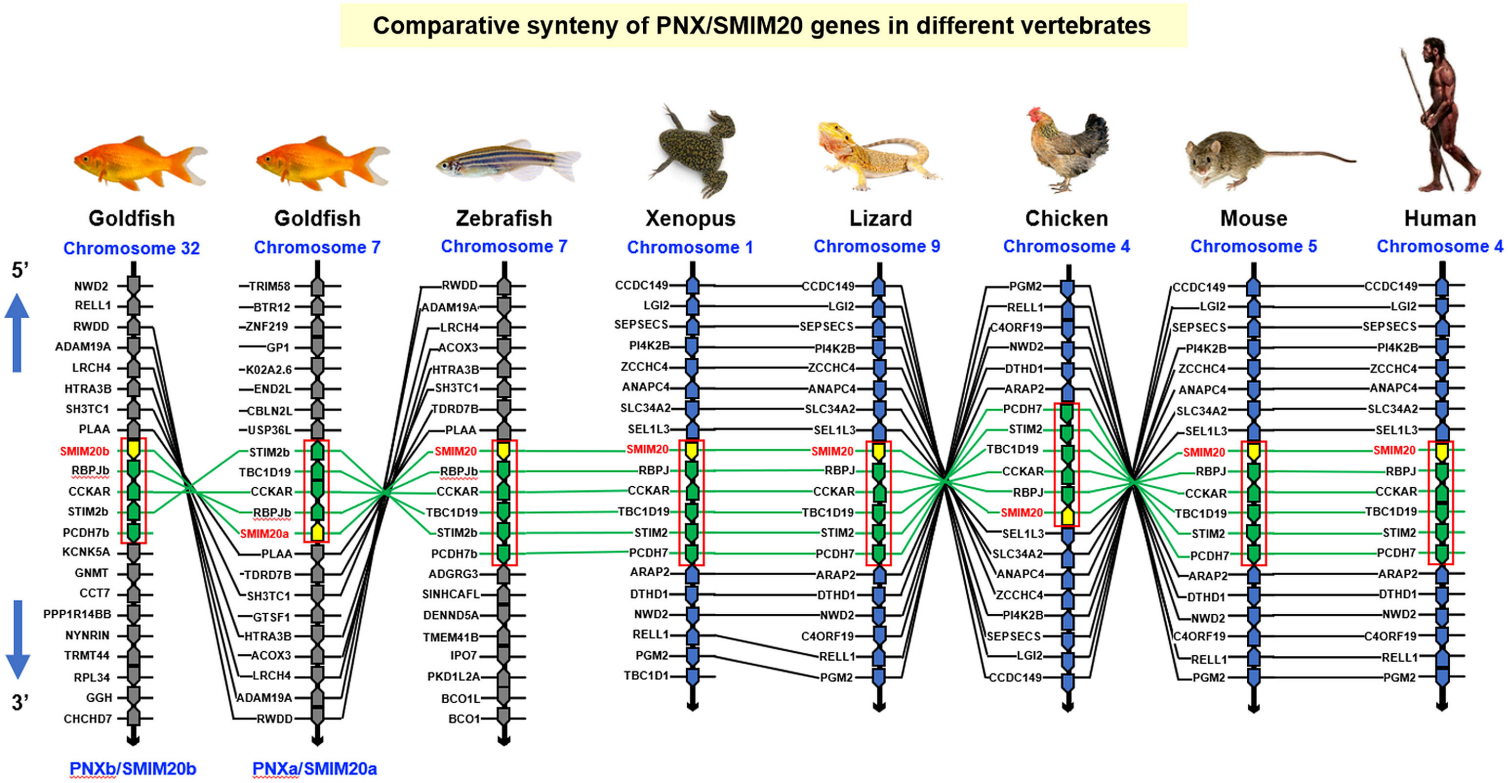
Comparative synteny of PNX/SMIM20 genes in different vertebrates. The genomic scaffolds with the SMIM20 gene in the respective chromosomes of representative species from different vertebrate classes were downloaded from the NCBI genome database. The neighboring genes located upstream and downstream of SMIM20 were annotated and curated using Genomicus. In selected segments within individual scaffolds, a well-conserved collinear syntenic block (marked with a red box) with SMIM20 followed by 4–5 associated genes (RBPJ, CCKAR, TBC1D19, STIM2, and PCDH7) in a fixed order can be identified across different species. Based on the similarity of syntenic genes around SMIM20 in the species examined, two clusters of evolution lineages related to SMIM20 can be recognized, including the fish lineage (goldfish and zebrafish) and tetrapod lineage (Xenopus, lizard, chicken, mouse, and human). Genomic translocation of the collinear synteny block with SMIM20 might have occurred during the evolution from fish to amphibians. The genes around SMIM20 in the same chromosomal segment are presented as polygons in a series with pointed ends indicating the transcriptional orientation. Across different species, the genes in the collinear syntenic block with SMIM20 are linked with green lines whereas the ortholog genes around the syntenic block in the fish and tetrapod lineages are linked with black lines.

To shed light on the evolutionary relationship of goldfish PNXa/SMIM20a and PNXb/SMIM20b with their counterparts in other vertebrates, phylogenetic analysis using the neighbor-joining method with MEGA X was performed in SMIM20 sequences identified in representative species of different vertebrate classes. As shown in **Supplementary Figure 3A**, the two forms of goldfish PNX/SMIM20 were clustered in the clade of fish SMIM20 and found to have a close evolutionary relationship with the SMIM20 in zebrafish (which is also a member of the cyprinids). Parallel analysis of the gene structure of SMIM20 in different species (including the 2 forms in goldfish) also revealed that the intron/exon organization of SMIM20 (with 2 introns and 3 exons) was well-conserved from fish to mammals (**Supplementary Figure 3B**). Interestingly, intron I of SMIM20 was found to exhibit a trend of size increase during vertebrate evolution (with the largest size observed in mammalian species). To study the evolution of the SMIM20 gene, microsyntenic analysis was performed in the neighboring genes around SMIM20 in species from different vertebrate classes. As shown in **Figure 2**, a conserved syntenic block with collinearity of RBPJ, CCKAR, TBC1D19, STIM2, and PCDH7 after the 3’ end of SMIM20 was identified in different chromosomal loci from fish to mammals (including the two newly cloned PNX in goldfish with deletion of PCDH7 and TBC1D19 in the syntenic blocks of PNXa/SMIM20a and PNXb/SMIM20b, respectively). Comparison of syntenic genes around SMIM20 also revealed two distinct lineages in vertebrate evolution, including (i) the fish lineage with SMIM20 surrounded by 10–13 collinear syntenic genes (e.g., goldfish and zebrafish) and (ii) the tetrapod lineage (from Xenopus to mammals) with SMIM20 surrounded by 18–19 collinear syntenic genes. Except for the 4–5 collinear genes in the syntenic block with SMIM20, the other syntenic genes around SMIM20 in the two lineages were found to be entirely different. These results imply that genomic translocation of the conserved syntenic block with SMIM20 followed by 4–5 associated genes in a fixed order might have occurred during the evolution from fish to the ancestor of amphibians.

**Figure 3:**
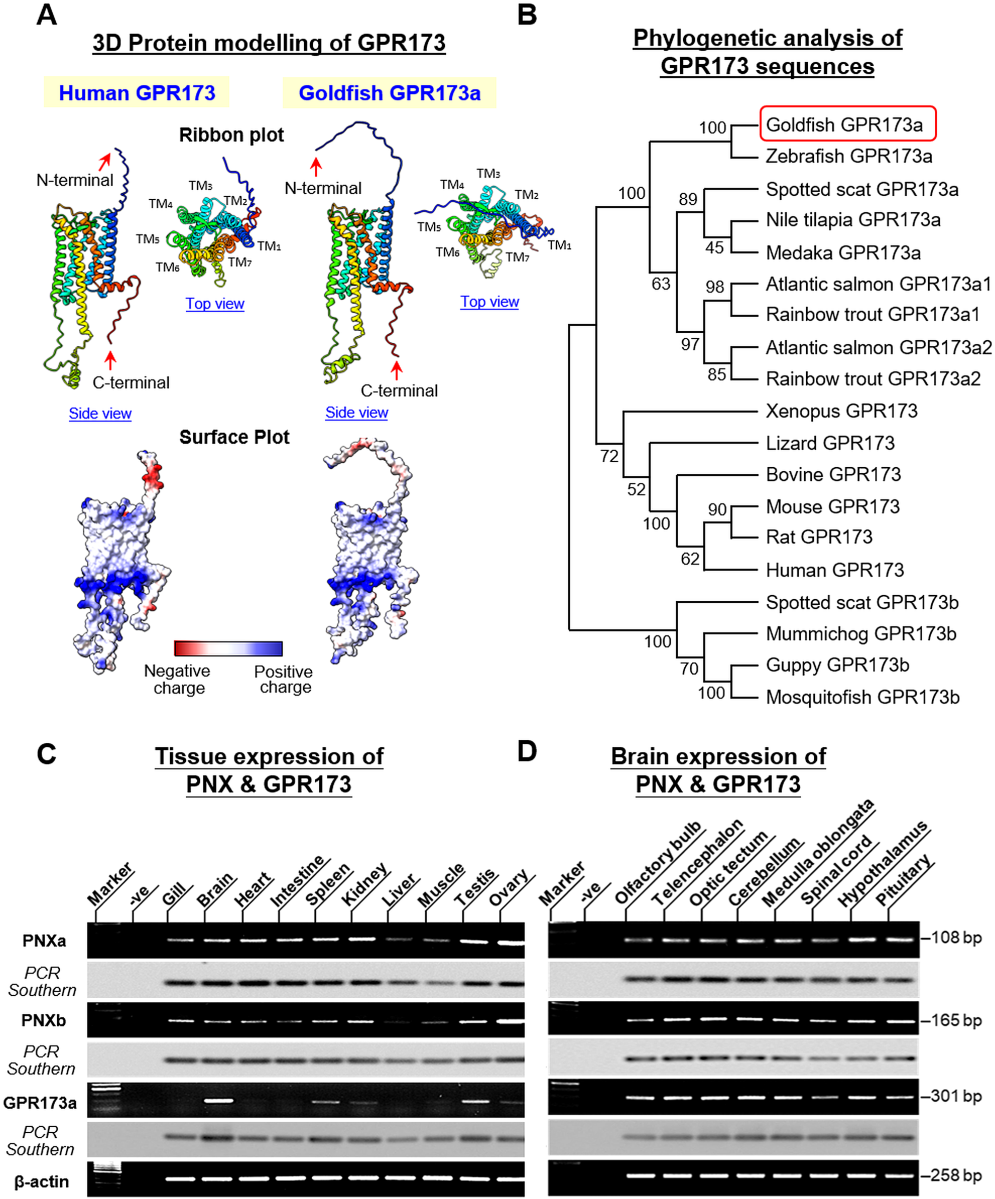
3D protein modeling and phylogenetic analysis of goldfish GPR173a and expression profiling of PNX/GPR173 system in different tissues and brain areas in goldfish. **(A)** *In silico* protein modeling of goldfish GPR173a with its human counterpart as the reference. 3D Protein modeling of GPR173 was constructed with AlphaFold and visualized using ChimeraX. The helical and random coil structures of deduced models are shown in the ribbon plots while the charge distribution on the molecular surface (blue for positive charge, red for negative charge, and white for non-polar residues) is presented in the surface plots. **(B)** Phylogenetic analysis of goldfish GPR173a with respect to its counterparts in representative species from different vertebrate classes using MEGA X with the neighbor-joining method. The guide tree was constructed using PHYLIP 2.0 with the percentage of bootstrap values (based on 1,000 bootstraps) shown in individual nodes. **(C, D)** RT-PCR for tissue expression profiling of PNXa, PNXb, and GPR173a in selected tissues and brain areas in goldfish. After agarose gel electrophoresis Southern blot with DIG-labelled probes for PNXa, PNXb, and GPR173a, respectively, was used to confirm the authenticity of PCR products. Parallel RT-PCR for β actin was also performed to serve as the internal control.

In fish models, two forms of GPR173, GPR173a and GPR173b, have been reported. GPR173a is more akin to the GPR173 found in tetrapods whereas GPR173b is expressed in some fish species (e.g., guppy and mosquitofish) but not others (e.g., zebrafish) (29). Using 5’/3’ RACE, the ORF of GPR173 (1164 bp in size) was also cloned in goldfish (**Supplementary Figure 4**). Structural analysis of the 387 a.a. protein product encoded by the newly cloned ORF confirmed that goldfish GPR173 was a member of GPR173a reported in fish models and with ×7 TMDs typical of the GPCR family. Alignment of goldfish GPR173a sequence with its counterparts in other species also revealed that the protein sequences of GPR173 were highly conserved from fish to mammals (with 84%–100% homology among vertebrate species) (**Supplementary Figure 5**). As shown in the 2D snake plot of goldfish GPR173a, the x7 TMDs (i.e., TMD_1-7_) formed helical structures and fitted into a hypothetical lipid bilayer. Except for the extracellular N-terminal with more variable sequences, TMD_1-7_ (with 82%–100% homology), extracellular loops 1–3 (ECL_1-3_) (with 73%–100% homology), intracellular loops 1–3 (ICL_1-3_) (with 93%–100% homology), and intracellular C-terminal (with 65%–100% homology) were found to be highly conserved among different species (**Supplementary Figure 6**). *In silico* protein modeling with human GPR173 as a reference also revealed that the TMD_1-7_ of goldfish GPR173a were all arranged in the form of α helixes and clustered together to form a “central pocket” (presumably acting as the binding site for PNX). Similar to human GPR173, the outer surface of these helical structures was found to be hydrophobic, which is supposed to allow the receptor to insert properly into the plasma membrane. Furthermore, the presence of acidic residues in the N-terminal and basic residues in the upper and lower boundaries of TMD_1-7_ were also highly comparable to their human counterparts (**Figure 3A**). To examine the phylogenetic relationship of goldfish GPR173a with respect to GPR173 in other species, MEGA X analysis with the neighbor-joining method was performed with the ORFs of GPR173 found in different vertebrate classes. Similar to the results of PNX analysis, goldfish GPR173a was clustered in the clade of GPR173a and had a close evolutionary relationship with its counterpart in zebrafish (**Figure 3B**). Although goldfish is a tetraploid fish and expected to have two isoforms of GPR173a (GPR173a1 and GPR173a2) similar to the cases of Atlantic salmon and rainbow trout, we could only extract one form of GPR173 (i.e., GPR173a) based on 5’/3’ RACE (the same was also true for our sequence search of GPR173a in goldfish and zebrafish databases). The reason why only a single form of GPR173a can be found in goldfish is still unknown.

Given that PNX and GPR173 are known to be widely expressed at the tissue level (e.g., in rodents) (1, 6), tissue expression profiling for PNXa, PNXb, and GPR173a was also conducted in goldfish using RT-PCR. As shown in **Figures 3C, D**, transcript signals for PNXa, PNXb, and GPR173a were found to be ubiquitously expressed in various tissues and brain areas. Among the tissues and organs examined, high levels of PNXa and PNXb expression were located in the ovaries followed by the testis and kidneys, to a lower extent in the gills, brain, heart, intestine, and spleen, and with relatively low levels in the liver and muscle. For the corresponding GPR173a signals, the highest level could be noted in the brain followed by the testis and spleen, and yet the levels of GPR173a expression in other tissues and organs were found to be much lower. Parallel RT-PCR in selected brain areas also revealed that PNXa, PNXb, and GPR173a were widely expressed within the CNS, including the olfactory bulb, telencephalon, hypothalamus, optic tectum, pituitary, cerebellum, medulla oblongata, and spinal cord (**Figure 3D**). High levels of PNXa signals were detected in the hypothalamus and pituitary, while the corresponding signals in other brain areas were lower. For PNXb signals, the telencephalon, hypothalamus, optic tectum, medulla oblongata, and pituitary were shown to have higher levels of expression but the signals in the olfactory bulb and spinal cord were notably weaker. Regarding GPR173a expression in the brain, despite lower levels of expression in the pituitary and spinal cord, high signal levels were found in the olfactory bulb, telencephalon, hypothalamus, optic tectum, cerebellum, and medulla oblongata.

### 3.2 Effect of food intake and food deprivation on PNXa, PNXb, and GPR173a expression in the liver and brain areas involved in appetite control in goldfish

**Figure 4:**
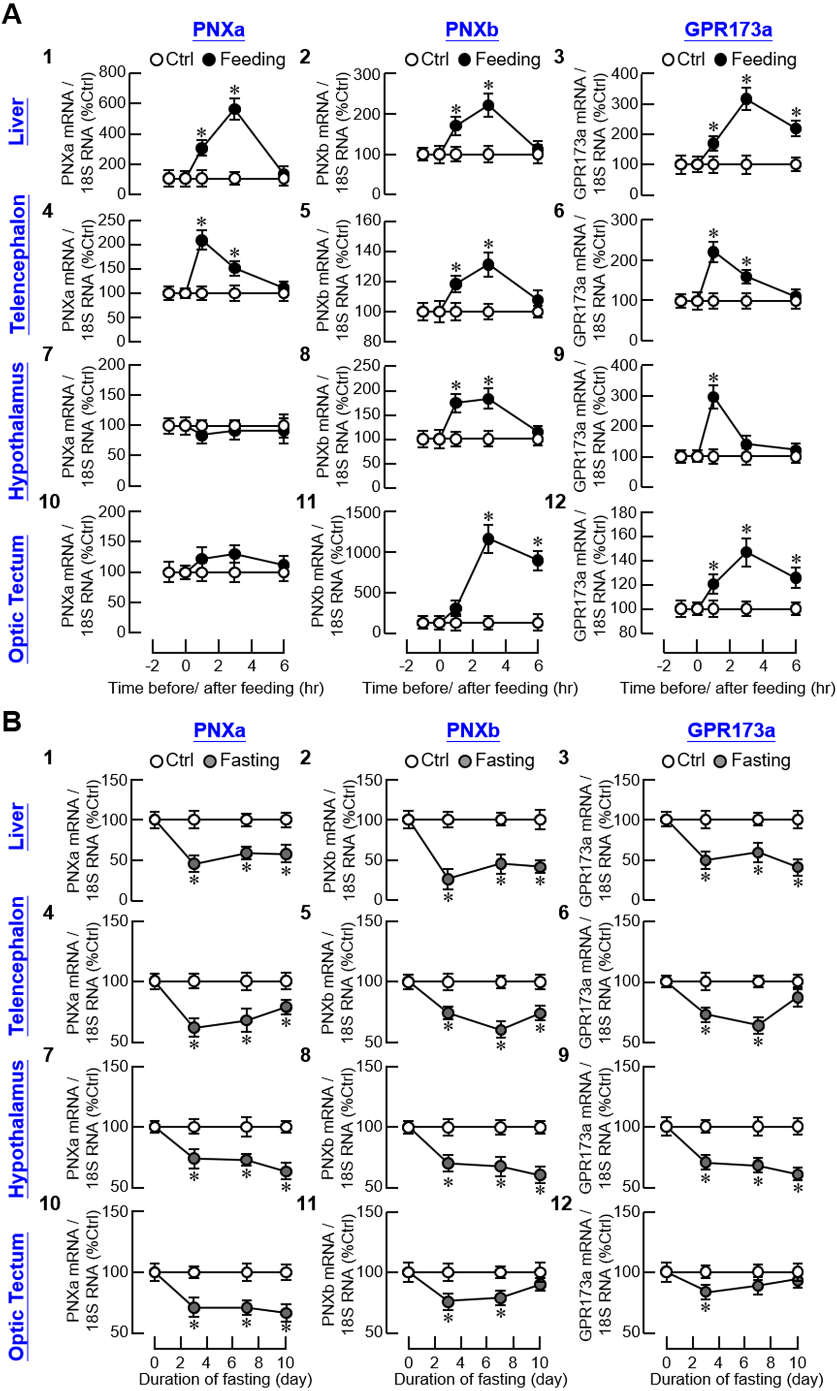
Effect of feeding status on the central and peripheral expression of PNXa/b and GPR173a in goldfish. **(A)** Food consumption on PNXa, PNXb, and GPR173a expression in the liver and brain areas including the telencephalon, hypothalamus and optic tectum. Goldfish were entrained with one-meal-per-day feeding schedule with food provision at 10:00 AM (taken as time zero) and tissue samples were harvested at the respective time points as indicated. Parallel group without food provision was used as the control. **(B)** Fasting on PNXa, PNXb and GPR173a expression in the liver, telencephalon, hypothalamus and optic tectum. After entraining with the one-meal-per-day feeding schedule, the fish were subjected to food deprivation up to 10 days with tissues harvested on the days as indicated. The fish with regular feeding was used as the control. In these studies, RNA samples isolated from the liver/different brain areas in individual groups (12 fish per group) were used for real-time PCR with primers for PNXa, PNXb, and GPR173a, respectively. Parallel real-time PCR for 18S RNA was also conducted to serve as the internal control. For the data presented, an asterisk (*) denotes a significant difference (*p* < 0.05) compared to the corresponding control.

To test for a functional link between the PNX/GPR173 system and feeding status in our animal model, goldfish entrained with a one-meal-per-day feeding regimen were subjected to food intake at 10:00 AM with the unfed group as control treatment. As shown in **Figure 4A**_1-3_, postprandial rises in PNXa, PNXb, and GPR173a transcripts with peak responses at 3 hr after the initiation of food intake were noted in the liver. In goldfish, the telencephalon, hypothalamus, and optic tectum are the major brain areas involved in appetite control (34). In our study, food intake induced transient elevations of PNXa, PNXb, and GPR173a transcripts in the telencephalon with peak responses occurring within the first 1–3 hr after food provision (**Figure 4A**_4-6_). Similar results were also obtained for PNXb and GPR173a transcripts expressed in the hypothalamus and optic tectum but notable changes in PNXa signals were not observed in these brain areas (**Figure 4A**_7-12_). Unlike the stimulatory responses for PNXa/b and GPR173a in the telencephalon (which could return to basal at 6 hr), the PNXb response in the optic tectum and GPR173a response in the liver and optic tectum were maintained after the peak phase (but with a lower magnitude) until the end of the 6-hr feeding experiment.

Regarding the reciprocal experiment for food intake, food deprivation up to 10 days was conducted in goldfish and the group with daily provision of food pellets at 10:00 AM was used as the control. In contrast to food intake, food deprivation was effective in reducing PNXa, PNXb, and GPR173a transcript levels in the liver and brain areas including the telencephalon, hypothalamus, and optic tectum (**Figure 4B**). In the liver, parallel drops in PNXa, PNXb, and GPR173a gene expression were noted on day 3 and maintained up to day 10 with fasting (**Figure 4B**_1-3_). With the exception of the PNXb response in the optic tectum and GPR173a responses in the telencephalon and optic tectum (which returned to basal on day 10), a similar pattern of sustained inhibition of PNXa/b and GPR173a signals was observed in the telencephalon (**Figure 4B**_4-6_), hypothalamus (**Figure 4B**_7-9_), and optic tectum (**Figure 4B**_10-12_).

### 3.3 Effect of IP and ICV injections of PNX20a and PNX20b on feeding behaviors and food consumption in goldfish

**Figure 5:**
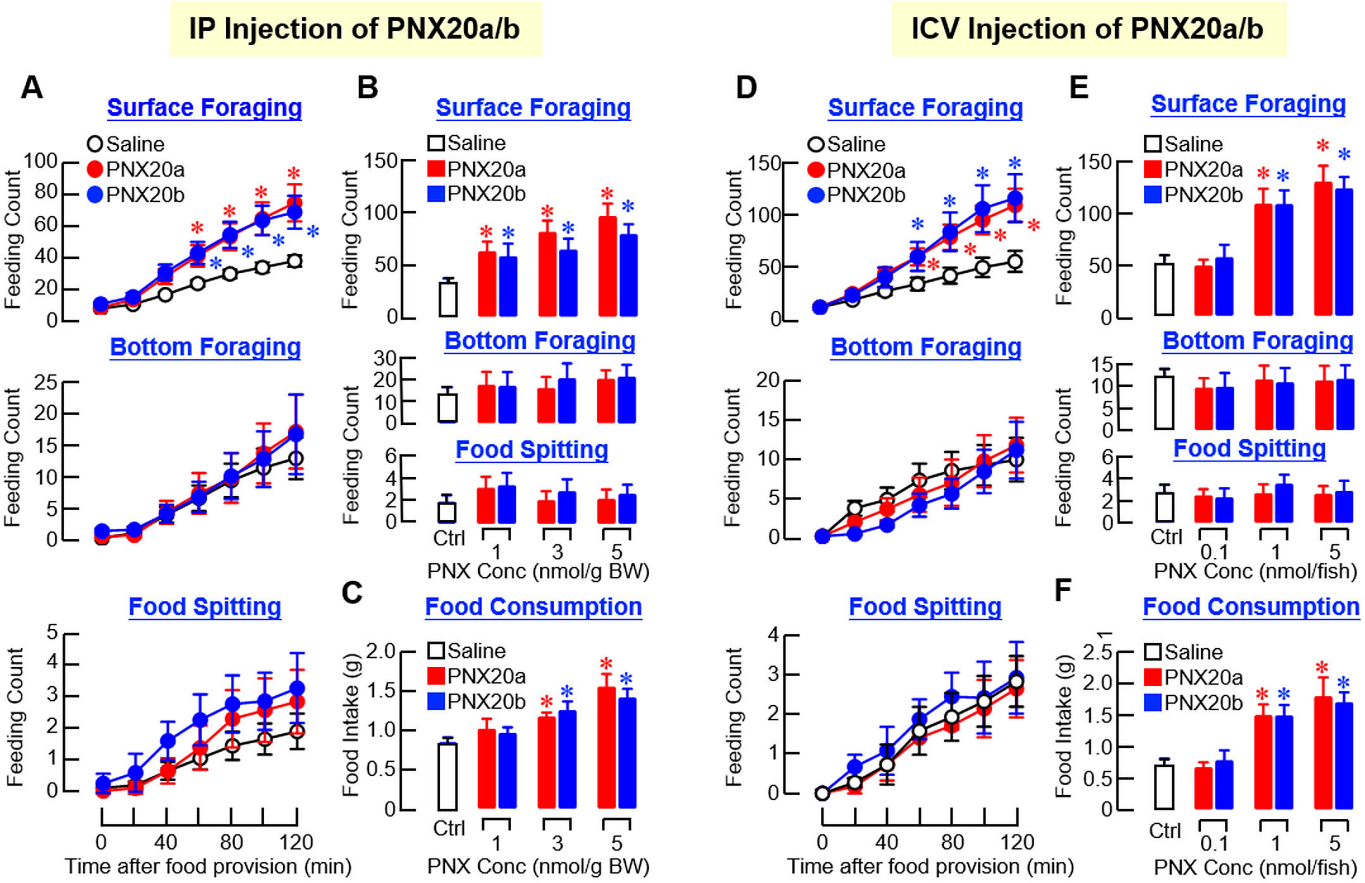
Effects of IP and ICV injections of PNX20a and PNX20b on feeding behaviors and food intake in goldfish. **(A)** Time course (3 nmol/g BW) and **(B)** dose dependence (1–5 nmol/g BW for 2 hr) for IP injections of PNX20a and PNX20b were conducted to test their effects on cumulative counts of surface foraging, bottom foraging, and food spitting in goldfish. In the study for dose dependence, food consumption for individual fish was also monitored **(C)**. For ICV injections of PNX20a and PNX20b, a similar approach for **(D)** time course (1 nmol/g BW) and **(E)** dose dependence of PNX20a/b treatment (0.1–5 nmol/g BW for 2 hr) on different types of feeding behaviors was also evaluated with parallel measurement of food consumption **(F)**. In these experiments (12 fish per group), parallel injection with physiological saline was used as the control. For the results presented, an asterisk (*) denotes a significant difference (*p* < 0.05) compared to the corresponding control.

To study the role of PNX in feeding control in our fish model, PNX20a and PNX20b, the mature peptides of PNXa and PNXb respectively, were synthesized and used for peripheral administration via IP injection to test their effects on feeding behaviors and food consumption in goldfish. In our study, IP injections (5 nmol/g BW) of PNX20a and PNX20b were both effective in elevating the cumulative counts of surface foraging in a time-dependent manner but with no effect on bottom foraging and food spitting (**Figure 5A**). By fixing the duration of drug treatment at 2 hr, IP injections of increasing levels of PNX20a or PNX20b (1–5 nmol/g BW) dose-dependently increased surface foraging (**Figure 5B**) with a parallel rise in food consumption (**Figure 5C**). In the same study, increasing doses of PNX20a/b did not alter the feeding counts of bottom foraging or food spitting. Given that drug treatment by IP injection cannot differentiate between the central actions of test substance via penetration of the blood-brain barrier (BBB) and its peripheral actions mediated by secondary signals involved in appetite control, central administration of PNX20a/b by ICV injection was also performed in our study. The approach was based on the stereotaxic setup and microinjection system for goldfish brain previously validated by Peter’s group (35) and has been used successfully in our recent study with goldfish to probe the central actions of adiponectin (AdipoQ) for feeding regulation (36). Similar to the results for IP injection, ICV injections (1 nmol/fish) of PNX20a and PNX20b respectively increased the feeding counts for surface foraging up to 2 hr in a time-dependent manner but with no effect on bottom foraging/food spitting (**Figure 5D**). In a parallel study with drug treatment fixed at 2 hr, ICV injections with increasing levels (0.1–5 nmol/fish) of PNX20a and PNX20b were both effective in increasing surface foraging (**Figure 5E**) with a parallel increase in food consumption in a dose-related fashion (**Figure 5F**). Again, no significant change was observed in this experiment in terms of bottom foraging/food spitting (**Figure 5E**).

### 3.4 IP and ICV injections of PNX20a and PNX20b on body motility and spatial preference for swimming in goldfish

**Figure 6:**
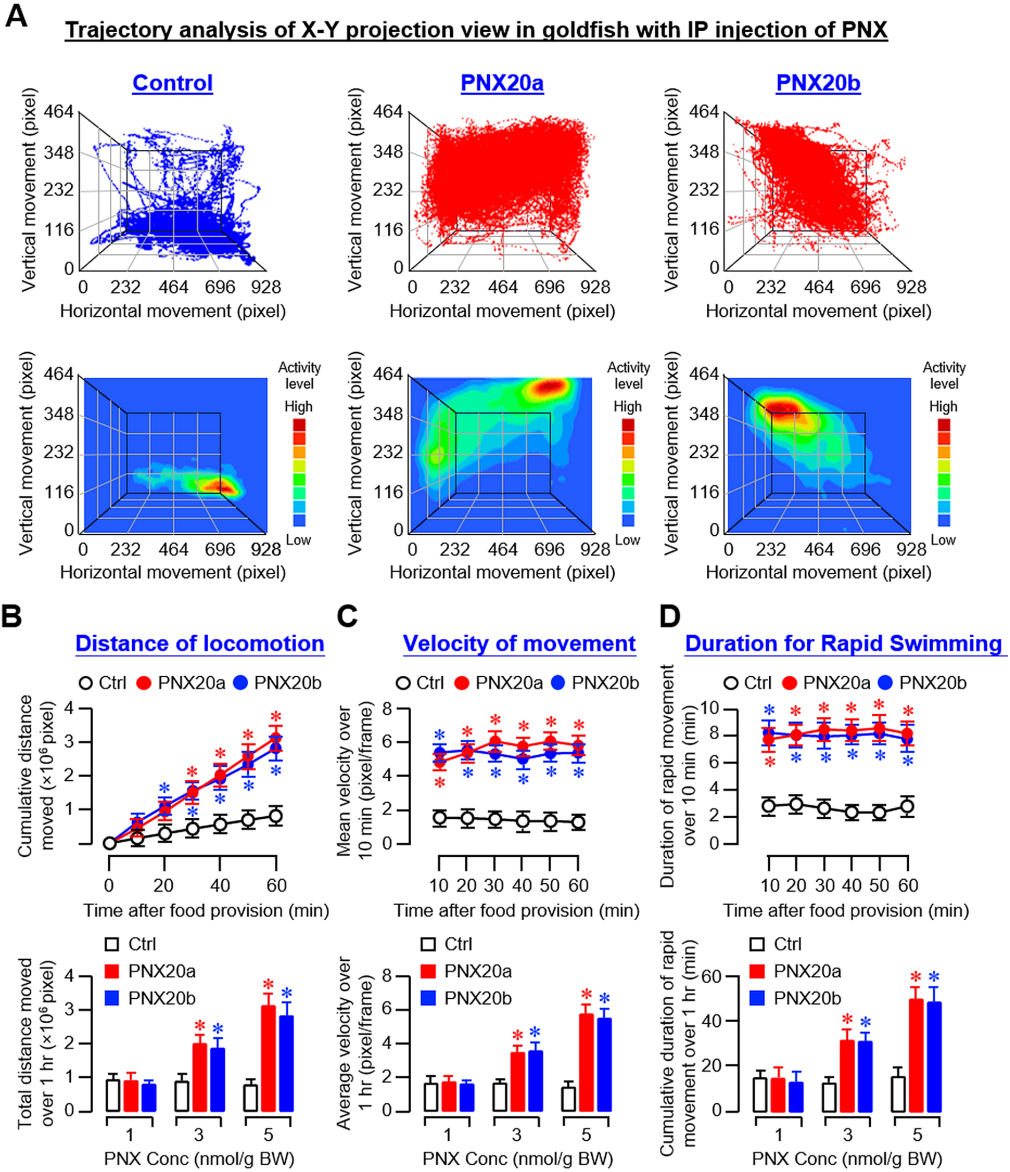
Analysis of body motility based on the X-Y projection view of trajectory traces of goldfish with IP injection of PNX20a/b. Body movement associated with feeding in goldfish (12 fish per group) with IP injection (5 nmol/g BW) of PNX20a/b was recorded for the duration as indicated with parallel injection of physiological saline as the control. The videos obtained were analyzed with DeepLabCut and coordinate data for vertical and horizontal movement with respect to time were used for the construction of trajectory plots and heat maps for motility assessment **(A)**. For quantitative analysis of body motility, cumulative distance of locomotion **(B)**, average velocity of movement **(C)**, and duration of fish engaged in rapid swimming **(D)** were deduced from the trajectory plots for time course study with IP injection (5 nmol/g BW) of PNX20a/b up to 1 hr (upper panels) and dose-dependence study with IP injection of increasing levels (1-5 nmol/g BW) of PNX20a/b (drug treatment for 1 hr, lower panels). For the data presented, an asterisk (*) denotes a significant difference (*p* < 0.05) compared to the corresponding control.

Based on the video recordings of our feeding experiments, trajectory analysis was conducted using DeepLabCut™ with supervised machine learning using a CNN model (33). Using the coordinate data generated, the trajectory plots and associated heatmaps derived from the X-Y projection view along the Z axis (i.e., the 3D plot of vertical and horizontal movement over time) revealed that the activity levels of locomotion in the groups with IP injections (5 nmol/g BW) of PNX20a and PNX20b were notably higher than that in the control (**Figure 6A**). Quantitative data extracted from trajectory plots also confirmed that PNX20a and PNX20b were both effective in increasing the locomotion distance (**Figure 6B**), velocity of movement (**Figure 6C**), and duration of rapid swimming (**Figure 6D**). To evaluate the spatial preference of locomotion after PNX treatment, coordinate data were also extracted from the Y-Z projection view of trajectory plots based on IP injection of PNX20a/b (with Y-axis for vertical movement and Z-axis for time). As shown in **Supplementary Figure 7A**, two discernible patterns of spatial preference were noted in the trajectory plots and heatmaps based on Y-Z projection views, including (i) the control group with locomotion occurring mainly in the lower half of the water body (also with occasional upward movements) and (ii) the groups with PNX20a/b treatment with high activity of locomotion in the upper half of the water body near the surface (also with frequent downward migration during the same period). Parallel analysis of the duration the fish spent in the upper half and lower half of the water body during the test period further confirmed the spatial preference of locomotion following PNX20 treatment. In this case, IP injections of PNX20a and PNX20b were both effective in increasing the duration of the goldfish staying in the upper half of the water in a time-dependent manner (and with a concurrent drop in the time spent in the lower half of the water during the same period) (**Supplementary Figure 7B**). Similar results for the dose-dependence of the responses in spatial preference were also noted in the parallel study with IP injections of increasing levels of PNX20a and PNX20b, respectively (**Supplementary Figure 7C**).

**Figure 7:**
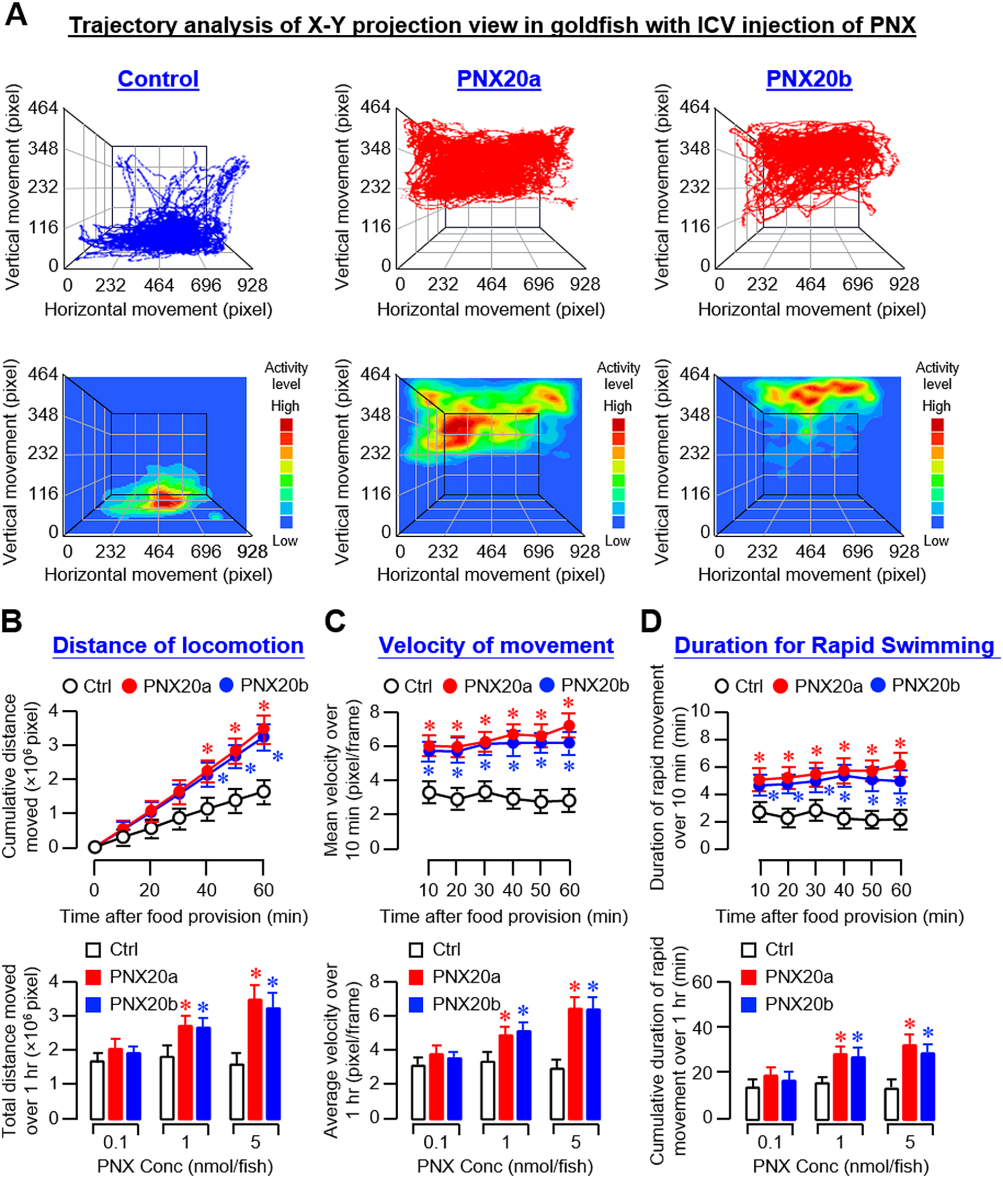
Analysis of body motility based on the X-Y projection view of trajectory traces of goldfish with ICV injection of PNX20a/b. Body movement associated with food intake in goldfish (12 fish per group) with ICV injection (5 nmol/g BW) of PNX20a/b was recorded for the duration as indicated with parallel injection of physiological saline as the control treatment. The videos obtained were analyzed with DeepLabCut and coordinate data for vertical and horizontal movement with respect to time were used for the construction of trajectory plots and heat maps for motility assessment **(A)**. For quantitative analysis of body movement, cumulative distance of locomotion **(B)**, average velocity of movement **(C)**, and duration of fish engaged in rapid swimming **(D)** were deduced from the trajectory plots for time course study with ICV injection (5 nmol/g BW) of PNX20a/b up to 1 hr (upper panels) and dose-dependence study with ICV injection of increasing levels (0.1–5 nmol/g BW) of PNX20a/b (drug treatment for 1 hr, lower panels). In the data presented, an asterisk (*) denotes a significant difference (*p* < 0.05) compared to the corresponding control.

For trajectory analysis based on videos taken during our feeding experiment with ICV injections (5 nmol/fish) of PNX20a and PNX20b, the results obtained were highly comparable with the preceding study based on IP injection. In this case, the trajectory plots and heatmaps of the X-Y projection view based on ICV injection revealed that the motility activity level in the group with PNX20a/b treatment was notably higher than that in the control group. Furthermore, the motility in the control group appeared to be concentrated near the bottom of the water tank whereas the groups treated with PNX20a/b were found to be more concentrated in the upper half of the water (**Figure 7A**). Quantitative analysis of the coordinate data based on trajectory plots also showed that ICV injection of PNX20a/b consistently increased the distance of locomotion (**Figure 7B**), velocity of movement (**Figure 7C**), and duration of rapid swimming up to 60 min (**Figure 7D**). As shown in the trajectory plots and heatmaps based on Y-Z projection views of the same study (**Supplementary Figure 8A**), the activity level of vertical movement in the control group was concentrated in the lower half of the water with occasional upward movements. In the groups with ICV injection of PNX20a/b (5 nmol/fish), the activity level of vertical movement was concentrated in the upper half of the water with frequent downward movements and notable motility (especially with PNX20b treatment) was located in the space under the water surface. Similar to the results based on IP injection, the cumulative time of the fish with ICV injection of PNX20a/b spent in the upper half of the water tank increased in a time- and dose-dependent manner up to 60 min with a concurrent drop in the time spent in the lower half of the water (**Supplementary Figures 8B, C**).

### 3.5 Effect of IP injections of PNX20a and PNX20b on appetite-regulating factors and their receptors expressed in brain areas involved in appetite control

**Figure 8:**
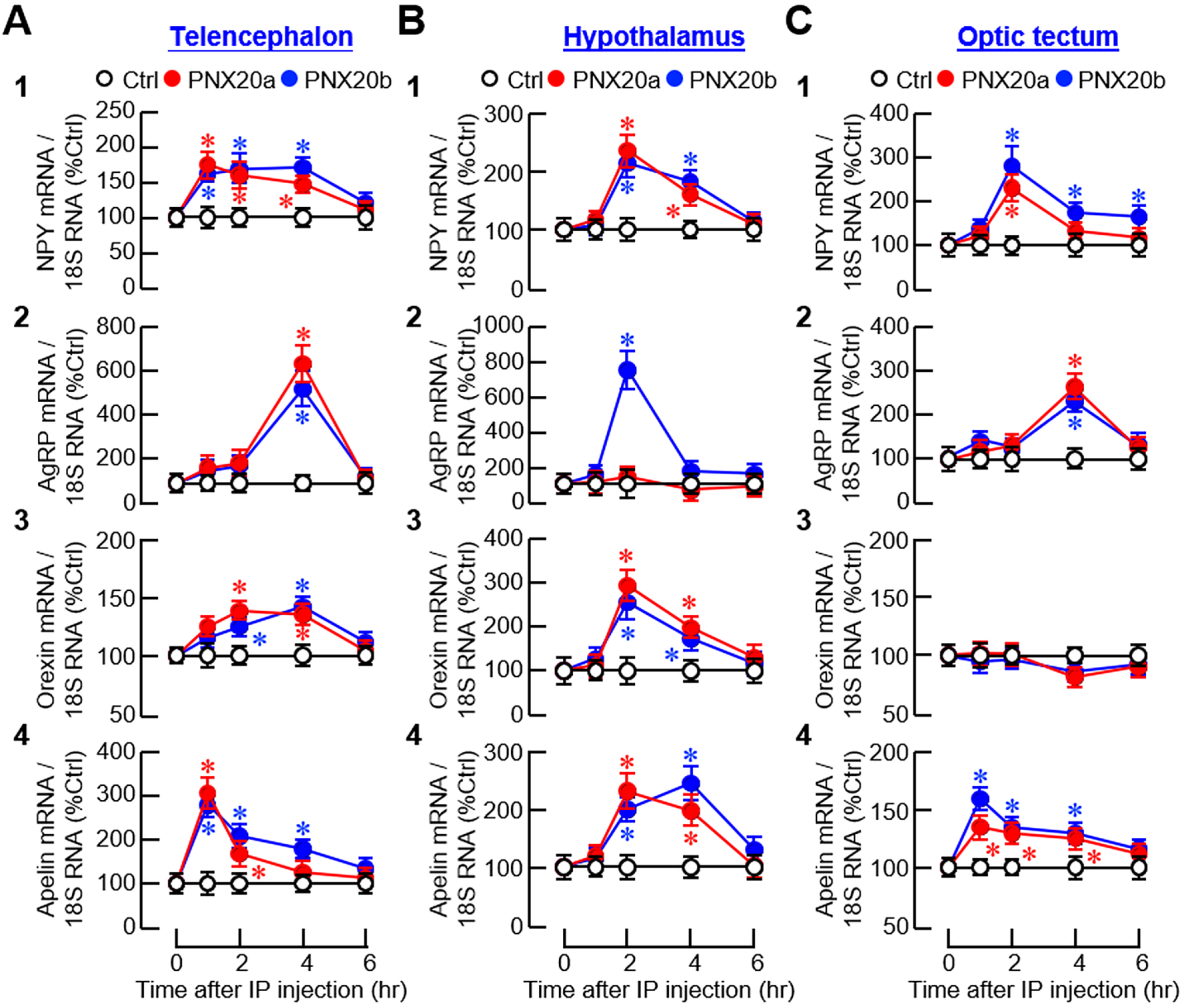
Effects of IP injection of PNX20a/b on orexigenic signals expressed in brain areas involved in appetite control. Goldfish (with 12 fish per group) were entrained with one-meal-per-day feeding schedule and subjected to IP injection (5 nmol/g BW) of PNX20a/b at 10:00 AM (taken as time zero). After that, brain areas including the telencephalon **(A)**, hypothalamus **(B)** and optic tectum **(C)** were harvested at the time points as indicated and used for total RNA isolation followed by real-time PCR for orexigenic factors including (1) NPY, (2) AgRP, (3) Orexin, and (4) Apelin. Parallel real-time PCR for 18S RNA was also conducted to serve as the internal control. For the data presented, an asterisk (*) represents a significant difference (*p* < 0.05) compared to its time-matched control.

To examine the mechanisms mediating PNX regulation of feeding, IP injections (3 nmol/g BW) of PNX20a and PNX20b were conducted in goldfish with subsequent monitoring of the appetite-regulating factors expressed in brain areas involved in feeding control. For central expression of orexigenic factors, except for the lack of responses for agouti-related peptide (AgRP) expression in the hypothalamus with PNX20a treatment (**Figure 8B**_2_) and orexin expression in the optic tectum with PNX20a/b treatment (**Figure 8C**_3_), IP injection of PNX20a/b was effective in triggering transient rises in NPY, AgRP, orexin, and apelin mRNA (with the peak response/plateau phase occurred during 1-4 hr) in the telencephalon (**Figure 8A**), hypothalamus (**Figure 8B**), and optic tectum (**Figure 8C**). Of note, the transient rises of orexigenic factors observed in the brain areas examined, except for NPY signals in the optic tectum with PNX20b treatment (**Figure 8C**_1_), all returned to basal 6 hr after drug treatment.

**Figure 9:**
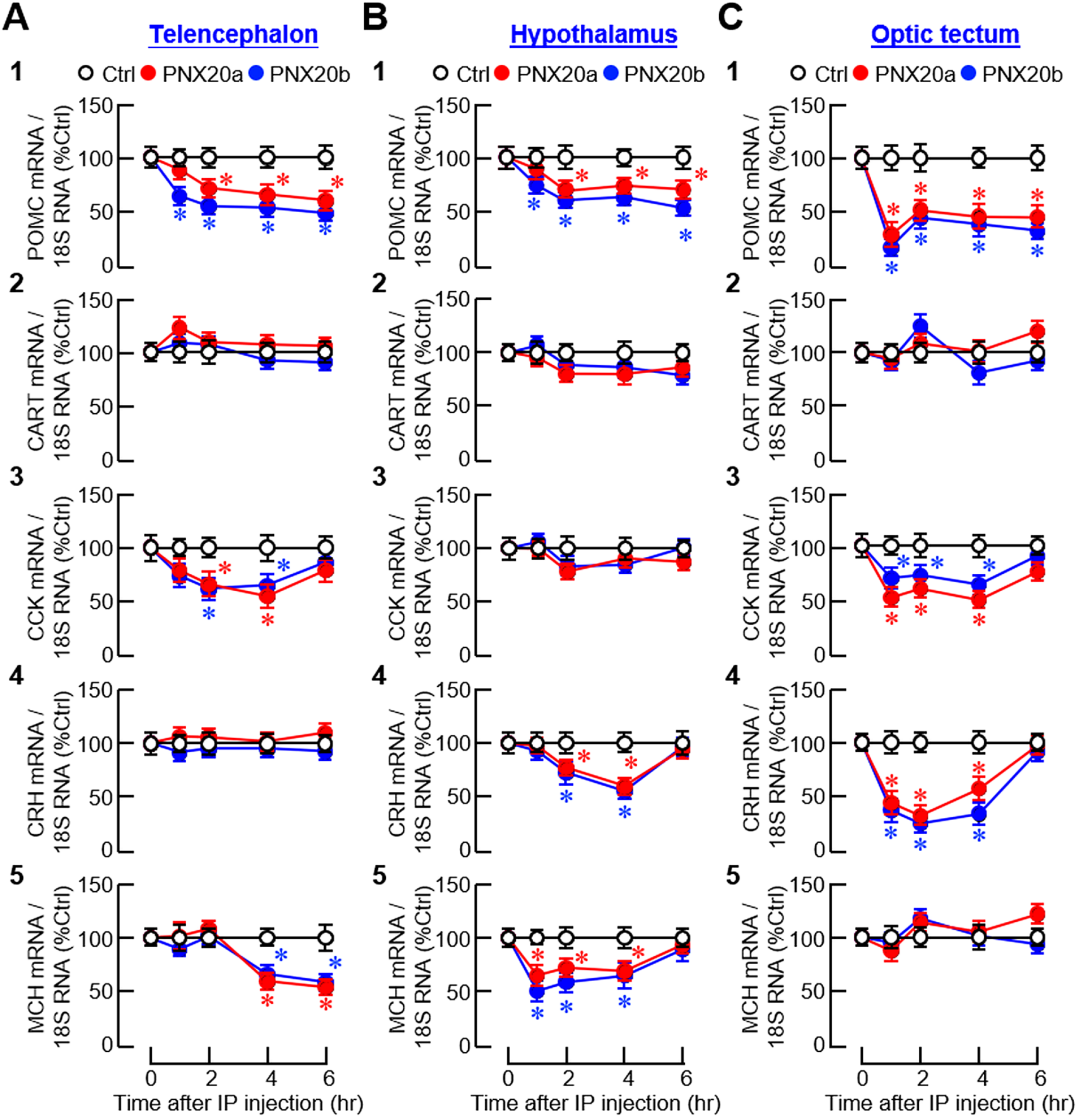
Effects of IP injection of PNX20a/b on anorexigenic signals expressed in brain areas involved in appetite control. Goldfish (with 12 fish per group) were subjected to IP injection (5 nmol/g BW) of PNX20a/b at 10:00 AM (taken as time zero). After that, brain areas including the telencephalon **(A)**, hypothalamus **(B)** and optic tectum **(C)** were harvested at the time points as indicated and used for total RNA isolation and real-time PCR for anorexigenic factors including (1) POMC, (2) CART, (3) CCK, (4) CRH, and (5) MCH. Real-time PCR for 18S RNA was used as the internal control. For the data presented, an asterisk (*) denotes a significant difference (*p* < 0.05) compared to its time-matched control.

For central expression of anorexigenic factors, IP injection of PNX20a/b notably reduced the transcript levels of pro-opiomelanocortin (POMC), CCK, and melanin-concentrating hormone (MCH) in the telencephalon (**Figure 9A**_1, 3 and 5_); POMC, corticotropin-releasing hormone (CRH), and MCH in the hypothalamus (**Figure 9B**_1, 4 and 5_); and POMC, CCK, and CRH in the optic tectum (**Figure 9C**_1, 3 and 4_). The same treatment, however, did not alter CRH expression in the telencephalon (**Figure 9A**_4_), CCK expression in the hypothalamus (**Figure 9B**_3_), MCH expression in the optic tectum (**Figure 9C**_5_), and CART expression in the three brain areas examined (**Figures 9A**_2_–**C**_2_). For the rapid inhibition observed during the first 1-2 hr after PNX20a/b treatment, two distinct patterns of gene expression were noted, including (i) sustained inhibition up to 6 hr for POMC expression in the telencephalon, hypothalamus, and optic tectum (**Figures 9A**_1_–**C**_1_), and (ii) transient inhibition for 3–4 hr with full recovery at 6 hr for CCK expression in the telencephalon and optic tectum (**Figures 9A**_3_, **C**_3_), CRH expression in the hypothalamus and optic tectum (**Figures 9B**_4_–**C**_4_), and MCH expression in the hypothalamus (**Figure 9B**_5_). During the course of PNX20a/b treatment, delayed inhibition starting at 4 hr and lasting up to 6 hr was also noted for MCH expression in the telencephalon (**Figure 9A**_5_).

**Figure 10:**
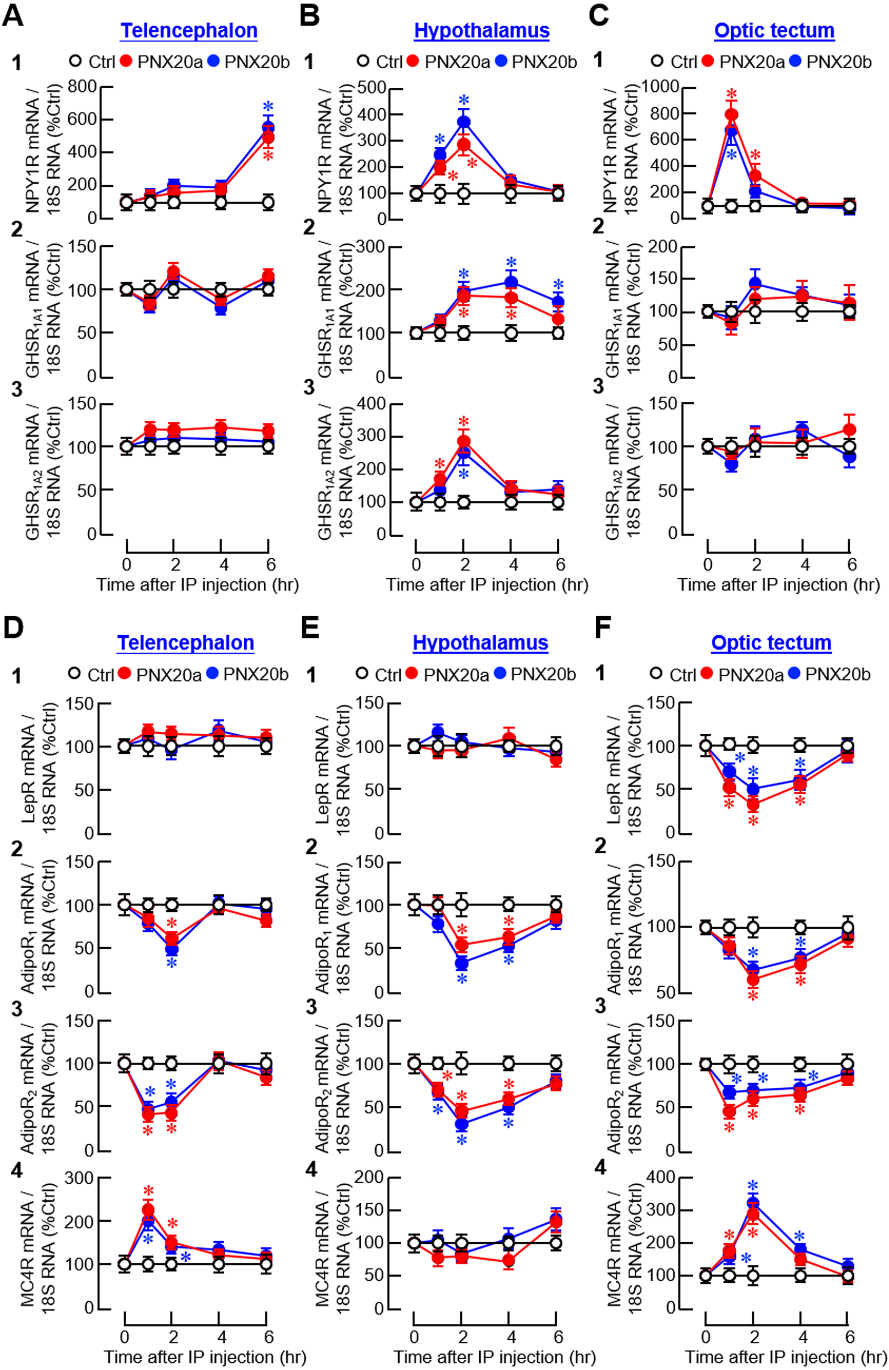
Effects of IP injection of PNX20a/b on receptors with orexigenic/anorexigenic actions expressed in brain areas involved in appetite control. Goldfish (with 12 fish per group) were subjected to IP injection (5 nmol/g BW) of PNX20a/b at 10:00 AM (taken as time zero). Brain areas including the telencephalon **(A, D)**, hypothalamus **(B, E)**, and optic tectum **(C, F)** were harvested at the time points as indicated. Following RNA isolation, real-time PCR was conducted for the receptors with orexigenic actions **(A-C)**, including (1) NPY1R, (2) GHSR _1A1_, and (3) GHSR_1A2_, as well as the receptors with anorexigenic actions **(D, E)**, including (1) LepR, (2) AdipoR_1_, (3) AdipoR_2_, and (4) MC4R. Real-time PCR for 18S RNA was used as the internal control. For the data presented, an asterisk (*) denotes a significant difference (*p* < 0.05) compared to its time-matched control.

Besides the feeding regulators examined, receptor expression for orexigenic (NPY and ghrelin) and anorexigenic factors (leptin, AdipoQ, and melanocortin) was also monitored to evaluate the sensitivity in selected brain areas for respective appetite-regulating signals. For the receptors of NPY (NPY1R) and ghrelin (GHSR_1A1_ and GHSR_1A_2__), despite a lack of response for ghrelin receptors in the telencephalon (**Figure 10A**_2-3_) and optic tectum (**Figure 10C**_2-3_), three distinct patterns of gene expression could be noted, including (i) a rapid but transient rise starting at 1 hr and with a peak occurred during the first 1-2 hr after PNX20a/b treatment followed by full recovery at 4 hr for NPY1R signals in the hypothalamus and optic tectum (**Figures 10B**_1_, **C**_1_) and GHSR_1A_2__ signals in the hypothalamus (**Figure 10B**_3_), (ii) a delayed increase starting at 2 hr for GHSR_1A1_ signals with a plateau phase maintained up to 4–6 hr in the hypothalamus (**Figure 10B**_2_), and (iii) a much-delayed increase of NPY1R signals occurring at 6 hr after PNX20a/b treatment in the telencephalon (**Figure 10A**_1_). For the receptor expression of leptin (LepR), AdipoQ (AdipoR_1_ and AdipoR_2_), and melanocortin (MC4R), except for the lack of responses for MC4R in the hypothalamus (**Figure 10E**_4_) and LepR in the telencephalon and hypothalamus (**Figure 10D**_1_, **E**_1_), a transient drop in transcript levels with a peak occurring 1–2 hr after PNX20a/b treatment followed by a full recovery to basal at 6 hr was detected for LepR expression in the optic tectum (**Figure 10F**_1_) and AdipoR_1_ and R_2_ expression in the hypothalamus (**Figure 10E**_2-3_) and optic tectum (**Figure 10F**_2-3_). Similar changes in AdipoR_1_ and R_2_ signals were also observed in the telencephalon but with an early recovery of the inhibitory responses at 4 hr after PNX20a/b treatment (**Figure 10D**_2-3_). Of note, unlike the inhibitory responses for LepR and AdipoR_1_/R_2_, PNX20a/b was effective in triggering a transient rise in MC4R expression with a peak occurring during the first 1–2 hr in the telencephalon and optic tectum after drug treatment (**Figures 10D_4_, F_4_**).

**Figure 11:**
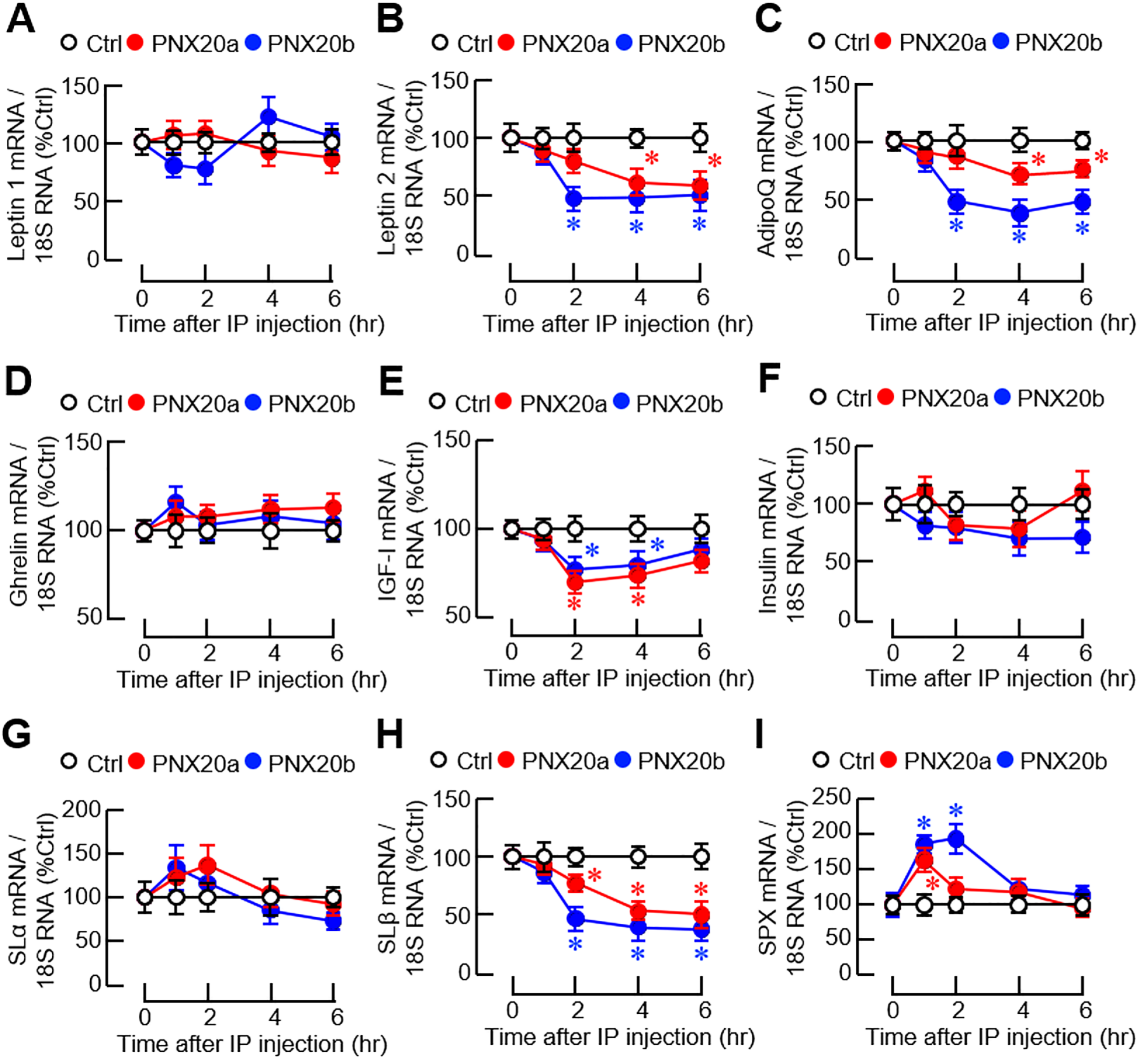
Effects of IP injection of PNX20a/b on appetite-regulating factors expressed in the liver. Goldfish (with 12 fish per group) were subjected to IP injection (5 nmol/g BW) of PNX20a/b at 10:00 AM (taken as time zero) and the liver was harvested at the time points as indicated. After total RNA isolation, real-time PCR was conducted for appetite-regulating factors including **(A)** Leptin 1, **(B)** Leptin 2, **(C)** AdipoQ, **(D)** Ghrelin, **(E)** IGF-I, **(F)** Insulin, **(G)** SLα, **(H)** SLβ, and **(I)** SPX. Real-time PCR for 18S RNA was used as the internal control. For the data presented, the group denoted by an asterisk (*) represents a significant difference (*p* < 0.05) compared to its time-matched control.

Given that the liver is known to be a source of endocrine signals with appetite-regulating functions in fish models, transcript expression of orexigenic (ghrelin) and anorexigenic factors [leptin 1/2, insulin, insulin-like growth factor-I (IGF-I), AdipoQ, spexin (SPX), and somatolactin (SL) α/β] was also monitored at the hepatic level. In our study, IP injection of PNX20a/b (3 nmol/g BW) did not alter leptin 1 (**Figure 11A**), ghrelin (**Figure 1D**), insulin (**Figure 11F**), and SLα expression in the liver (**Figure 11G**). However, downregulation of leptin 2 (**Figure 11B**), AdipoQ (**Figure 11C**), IGF-I (**Figure 11E**), and SLβ transcript expression (**Figure 11H**) was noted 2–4 hr after PNX20a/b treatment. Except for the IGF-I response with a full recovery at the end, the inhibition of leptin 2, AdipoQ, and SLβ expression was maintained up to 6 hr. Interestingly, unlike the inhibitory actions for leptin 2, AdipoQ, IGF-I, and SLβ signals, transcript expression of SPX was found to exhibit a transient rise with a peak/plateau phase occurring 1–2 hr after PNX20a/b treatment (**Figure 11I**).

### 3.6 ICV injections of PNX20a and PNX20b on appetite-regulating factors and their receptors expressed in brain areas involved in appetite control

**Figure 12:**
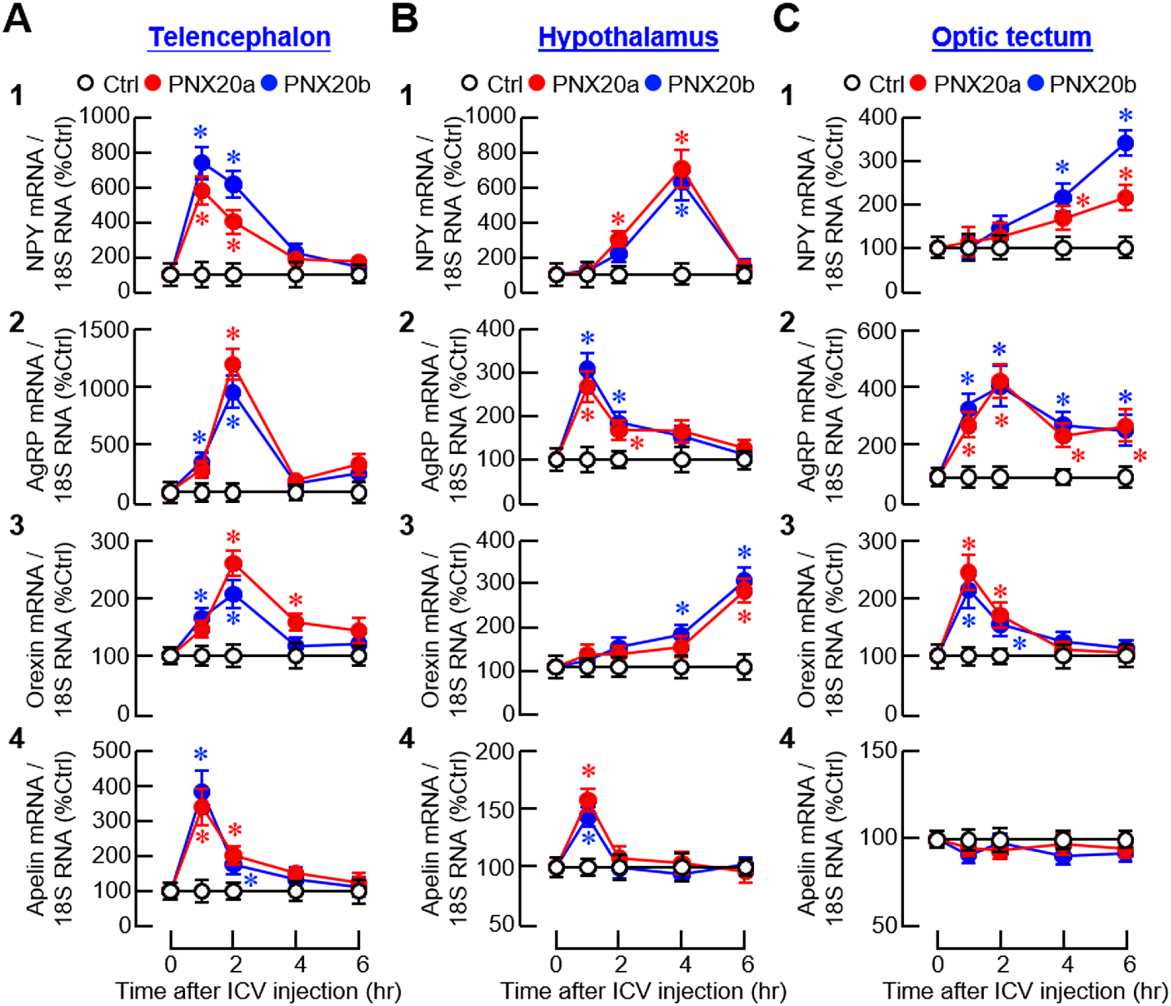
Effects of ICV injection of PNX20a/b on orexigenic signals expressed in brain areas involved in appetite control. Goldfish (12 fish per group) entrained with a one-meal-per-day feeding schedule were subjected to ICV injection (5 nmol/g BW) of PNX20a/b at 10:00 AM (taken as time zero). Brain areas including the telencephalon **(A)**, hypothalamus **(B)**, and optic tectum **(C)** were harvested at time points as indicated and used for total RNA isolation and real-time PCR for orexigenic factors including (1) NPY, (2) AgRP, (3) Orexin, and (4) Apelin. Real-time PCR for 18S RNA was used as the internal control. For the data presented, the group denoted by an asterisk (*) represents a significant difference (*p* < 0.05) compared to its time-matched control.

To confirm the central actions of PNX on feeding regulation, orexigenic/anorexigenic factors and their receptors expressed in the brain areas for appetite control were monitored in goldfish with ICV injections (1 nmol/fish) of PNX20a and PNX20b, respectively. For the expression of orexigenic factors, except for a lack of response for apelin expression in the optic tectum (**Figure 12C**_4_), PNX20a and PNX20b were both effective in increasing NPY, AgRP, orexin, and apelin transcript levels in the telencephalon, hypothalamus, and optic tectum (**Figures 12A–C**). For the responses observed, three discernible patterns of gene expression could be noted after PNX20a/b treatment, including (i) a transient rise with a peak occurred during 1–4 hr followed by full recovery at the end for NPY, AgRP, orexin, and apelin signals in the telencephalon (**Figure 12A**_1-4_); NPY, AgRP, and apelin signals in the hypothalamus (**Figure 12B**_1, 2 and 4_); and orexin signals in the optic tectum (**Figure 12C**_3_), (ii) a notable increase with a peak at 2 hr followed by a plateau with lower magnitude up to 6 hr for AgRP expression in the optic tectum (**Figure 12C**_2_), and (iii) a delayed stimulation with gradual rise observed during 4–6 hr for orexin expression in the hypothalamus (**Figure 12B**_3_) and NPY expression in the optic tectum (**Figure 12C**_1_).

**Figure 13:**
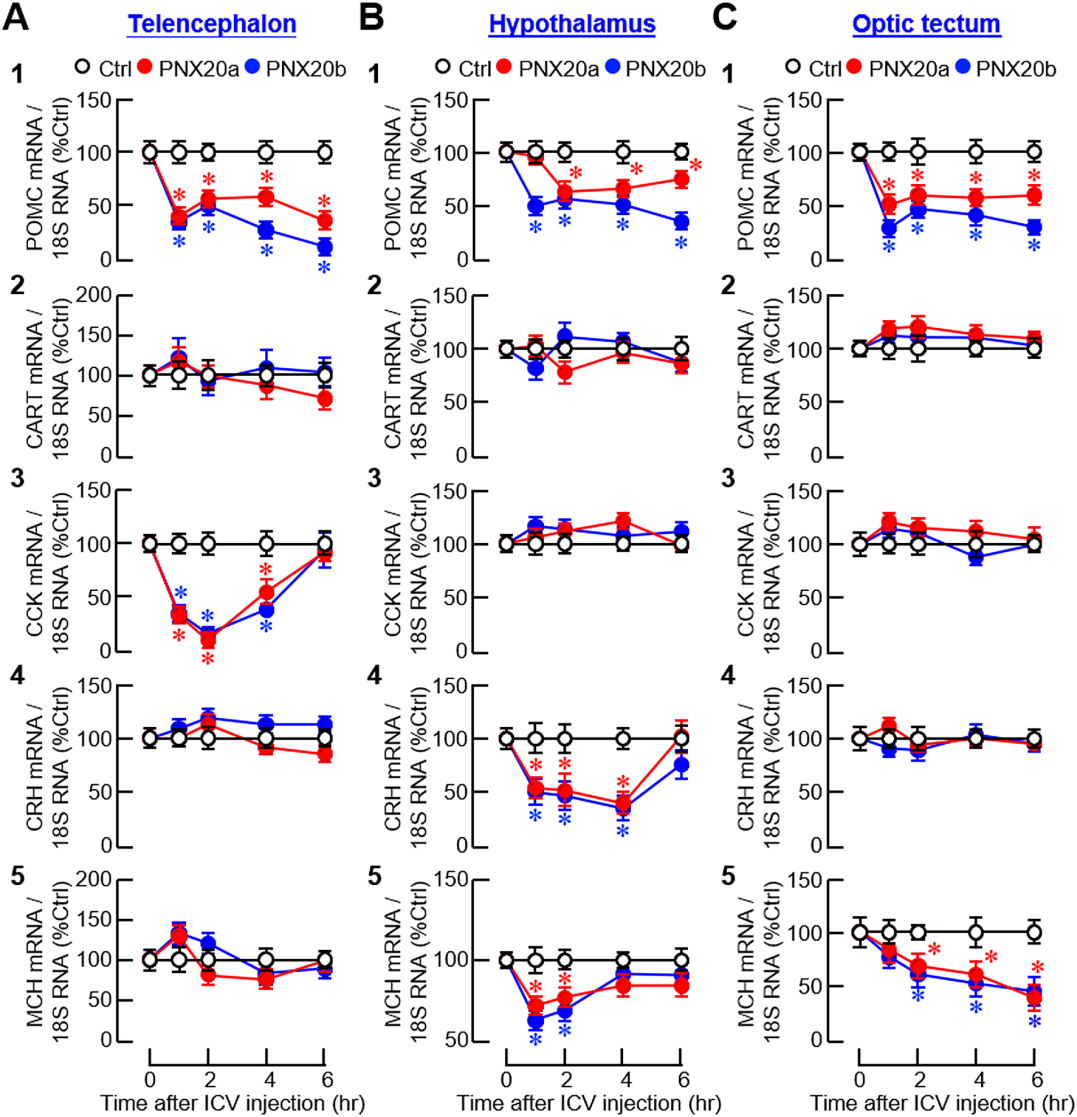
Effects of ICV injection of PNX20a/b on anorexigenic signals expressed in brain areas involved in appetite control. Goldfish (12 fish per group) were subjected to ICV injection (5 nmol/g BW) of PNX20a/b at 10:00 AM (taken as time zero). After that, brain areas including the telencephalon **(A)**, hypothalamus **(B)** and optic tectum **(C)** were harvested at the time points as indicated and used for RNA isolation followed by real-time PCR for anorexigenic factors including (1) POMC, (2) CART, (3) CCK, (4) CRH, and (5) MCH. Real-time PCR for 18S RNA was used as the internal control. For the data presented, the group denoted by an asterisk (*) represents a significant difference (*p* < 0.05) compared to its time-matched control.

For the responses of anorexigenic factors, notable inhibition was observed for POMC, CCK, CRH, and MCH mRNA levels in different brain areas after ICV injection of PNX20a/b (**Figure 13**). In this case, two distinct patterns of gene expression were noted, including (i) a rapid and sustained inhibition started during the first 1-2 hr and maintained up to 6 hr for POMC signals in the telencephalon and hypothalamus (**Figures 13A**_1_, **B**_1_) and POMC and MCH signals in the optic tectum (**Figure 13C**_1_, **C**_5_); and (ii) a rapid but transient drop started at 1 hr followed by a full recovery in 4–6 hr for CCK expression in the telencephalon (**Figure 13A**_3_) and CRH and MCH expression in the hypothalamus (**Figure 13B**_4-5_). In the same experiment, PNX20a/b treatment did not alter CART, CRH, and MCH signals in the telencephalon (**Figure 12A**_2, 4 and 5_); CART and CCK signals in the hypothalamus (**Figure 12B**_2-3_); and CART, CCK, and CRH signals in the optic tectum (**Figure 12C**_2-4_).

**Figure 14:**
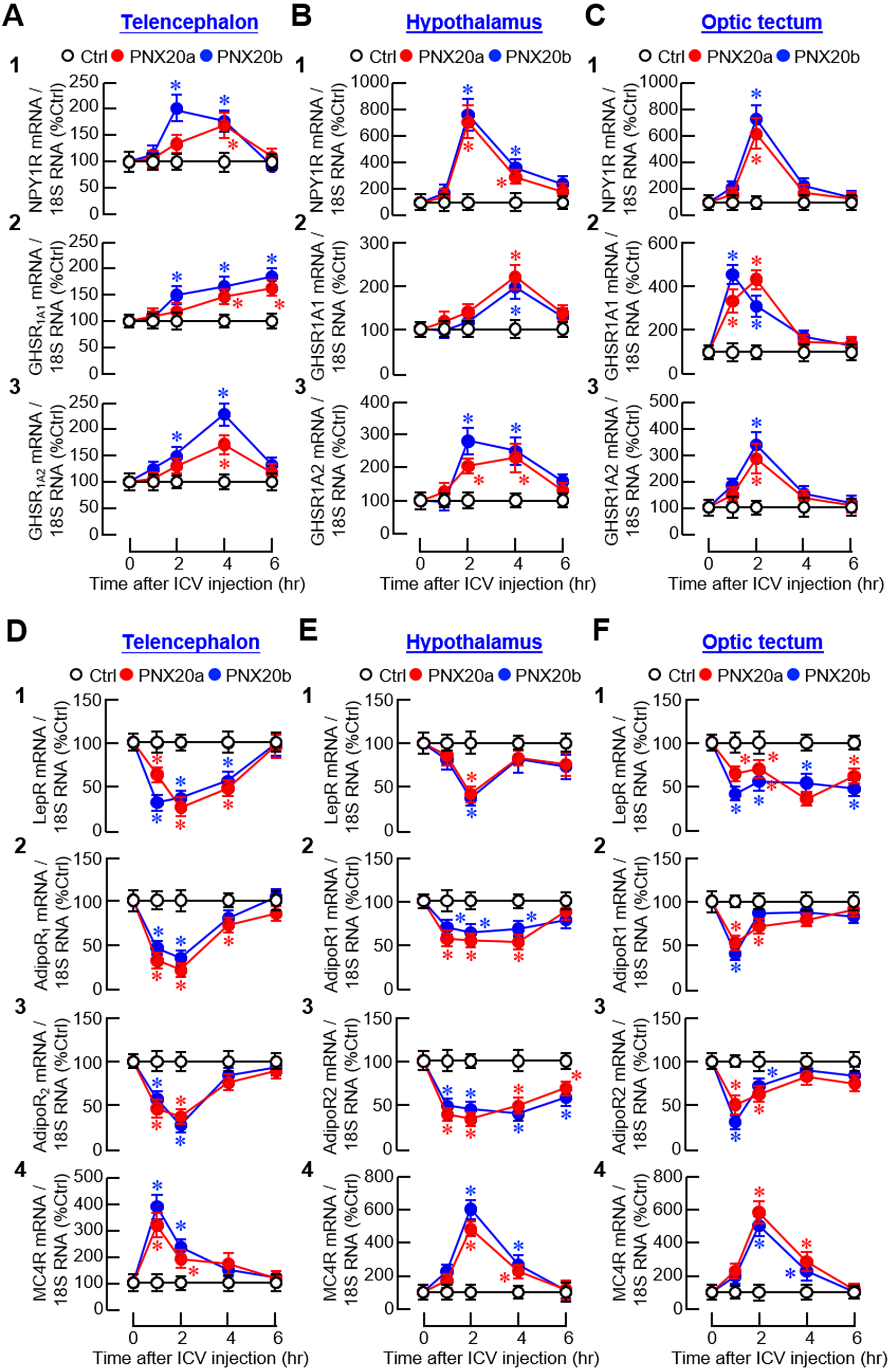
Effects of ICV injection of PNX20a/b on receptors with orexigenic/anorexigenic actions expressed in brain areas involved in appetite control. Goldfish (12 fish per group) were subjected to ICV injection (5 nmol/g BW) of PNX20a/b at 10:00 AM (taken as time zero). After that, brain areas including the telencephalon **(A, D)**, hypothalamus **(B, E)**, and optic tectum **(C, F)** were harvested at time points as indicated. Following total RNA isolation, real-time PCR was conducted for **(A-C)** the receptors with orexigenic actions, including (1) NPY1R, (2) GHSR1A1 _1A1_, and (3) GHSR_1A2_, as well as **(D, E)** the receptors with anorexigenic actions, including (1) LepR, (2) AdipoR_1_, (3) AdipoR_2_, and (4) MC4R. Real-time PCR for 18S RNA was used as the internal control. For the data presented, the group denoted by an asterisk (*) represents a significant difference (*p* < 0.05) compared to its time-matched control.

For the receptors with orexigenic actions, transcript levels of the receptors for NPY (NPY1R) and ghrelin (GHSR_1A1_/GHSR_1A2_) were upregulated in brain areas involved in appetite control after ICV injection of PNX20a/b (**Figure 14A**). Except for the gradual rise in GHSR_1A1_ expression up to 6 hr observed in the telencephalon (**Figure 14A**_2_), NPY1R, GHSR_1A1_, and GHSR_1A2_ signals all increased to their respective peaks during 1–4 hr and with full recovery at 6 hr in the telencephalon (**Figure 14A**_1_, **A**_3_), hypothalamus (**Figure 14B**_1-3_), and optic tectum (**Figure 14C**_1-3_). For the receptors with anorexic actions, PNX20a/b was shown to have differential effects on the receptors for leptin (LepR), AdipoQ (AdipoR_1_ and R_2_), and melanocortin (MC4R) in the three brain areas examined. For the responses observed, three distinct patterns of gene expression were recognized, including (i) a rapid but transient drop with a peak/plateau phase during 1–4 hr followed by full recovery at the end for LepR, AdipoR_1_, and AdipoR_2_ signals in the telencephalon (**Figure 14D**_1-3_); LepR and AdipoR_1_ signals in the hypothalamus (**Figure 14E**_1-2_); and AdipoR_1_ and AdipoR_2_ signals in the optic tectum (**Figure 14F**_2-3_), (ii) a rapid reduction starting at 1 hr and sustained up to 6 hr for AdipoR2 signals in the hypothalamus (**Figure 14E**_3_) and LepR signals in the optic tectum (**Figure 14F**_1_), and (iii) a transient rise with a peak during 1–2 hr followed by full recovery in 4–6 hr for MC4R expression in the telencephalon (**Figure 14D**_5_), hypothalamus (**Figure 14E**_5_), and optic tectum (**Figure 14E**_5_).

## 4 Discussion

PNX20, the mature peptide of SMIM20, is known to have pleiotropic functions in different tissues (see Introduction). Its functional role in appetite control varies among different species and a common consensus is still lacking. In higher vertebrates, including rats (10) and chickens (26), ICV injection of PNX14, a truncated peptide of PNX20, is known to induce food intake. In zebrafish, however, IP injection of PNX20 can lead to the opposite effect with inhibition of food consumption (27), suggesting that feeding regulation by PNX may differ in lower vertebrates. Given that only two reports have been published in fish related to PNX and feeding, with one in zebrafish (27) and the other in spotted scat (28), and the results of which are contradictory for PNX expression caused by fasting (see Introduction), a systematic study was initiated with goldfish as a model to examine the functional role and underlying mechanisms for feeding control by PNX in lower vertebrates. As a first step, two forms of PNX (PNXa/SMIM20a and PNXb/SMIM20b) and one form of GPR173a were cloned in goldfish. The two forms of PNX have been confirmed to be originated from separate genes with similar intron/exon organization in different chromosomes of the goldfish genome (with PNXa in chromosome 7 and PNXb in chromosome 32). The two PNX genes identified are probably the result of whole-genome duplication that occurred during the evolution of cyprinid species (37). As revealed by sequence alignment and phylogenetic analysis, goldfish PNXa/b and GPR173a are highly homologous to their counterparts in different species and can be clustered in the clades of fish PNX and GPR173a, respectively. Our *in silico* protein modeling also confirms that the 3D protein structures for goldfish PNXa, PNXb, and GPR173a in terms of surface charge distribution and spatial orientation of subdomain structures are highly comparable if not identical to their counterparts in humans. Furthermore, the 3D models for goldfish PNXa and PNXb with a highly conserved TMD motif imply that the PNX precursor SMIM20 may exist as a transmembrane protein. Although the shedding mechanism is still unknown, the mature peptides (i.e., PNX20a and PNX20b) are supposed to be released from the C-terminal by protein processing via the mono/dibasic cleavage sites flanking at both ends. Our idea is consistent with a previous report in human cell lines (e.g., HEK293 and U2OS cells), in which SMIM20 was found to be a mitochondrial membrane protein and involved in COX1 stabilization by forming the MITRAC complex during cytochrome c oxidase assembly (38). For goldfish GPR173a, the highly conserved a.a. sequence, the unique pattern of TMD_1–7_ clusterings, and the spatial arrangement of ECL_1–3_, ICL_1–3_, and the N/C-terminals indicate that it is a member of GPCR family. As a matter of fact, structural analysis of previous studies on GPR173 of mammalian origin has classified the receptor as a typical member of the rhodopsin-like class A GPCR (21, 22). In fish models, unlike a single form of GPR173 in mammals, two isoforms of GPR173, GPR173a and GPR173b, can been identified (29, 30). Recently, differential expression of GPR173a and GPR173b has been reported in the hypothalamo-pituitary axis of spotted scat at different stages of gonadal maturation, suggesting that the two forms may have different functions in reproduction (30). Interestingly, GPR173a and GPR173b are also differentially expressed in different orders of bony fish during evolution, e.g., the two forms of receptor can be found in tetradontiformes, cichliformes, and beloniformes, while GPR173a and GPR173b have been lost in cyprinodontiformes and cypriniformes, respectively (29). Given that goldfish is a member of cypriniformes, this may explain why only GPR173a (but not GPR173b) could be identified in our cloning study.

Given that the a.a. sequences of PNX and its putative receptor GPR173 are highly conserved in vertebrates, it is likely that the PNX/GPR173 system was evolved under a strong selection pressure and probably involved in important functions essential for survival. To shed light on the evolution of PNX gene, comparative synteny and structural organization analysis were conducted with PNX/SMIM20 genes in representative species from different vertebrate classes. Our results reveal that the structural organization of the genes coding for PNX/SMIM20 is well-conserved in vertebrates and composed of three exons and two introns from fish to mammals. Of note, the syntenic environment downstream of the 3’ end of the PNX/SMIM20 gene is well-conserved and consistently associated with 4–5 genes in a fixed order (including RBPJ, CCKAR, TBC1D19, STIM2, and PCDH7). Although the syntenic genes upstream of the 5’ end of PNX/SMIM20 are also highly comparable in fish species (e.g., in zebrafish and goldfish), the syntenic genes upstream/downstream of the collinear syntenic block containing PNX/SMIM20 and the 4–5 associated genes are entirely different from those in fish models but well conserved in tetrapods (e.g., in Xenopus, lizards, chickens, rats, and humans). These findings indicate that the syntenic block with PNX/SMIM20 and the 4–5 collinear genes in fish species had been relocated to a different locus within the genome during the evolution of amphibians. To our knowledge, our study represents the first to report the genomic translocation of the PNX/SMIM20 gene during the land invasion by vertebrates.

In mammals (e.g., rats), high levels of PNX immunoreactivity can be located in the heart and hypothalamus (1) while PNX transcripts are widely expressed in different brain areas and peripheral tissues (1, 6). For tissue distribution of GPR173, its transcripts are highly expressed in the brain (21, 22) and ovaries (39, 40), and to a lower extent in the small intestine (21). In fish models, including zebrafish (27) and spotted scat (28), transcript signals of PNX can be recognized in the hypothalamus and in a wide range of peripheral tissues. Except for the report in zebrafish (27) showing a strong signal of GPR173 transcript in the brain (especially in the hypothalamus) together with weaker signals in other tissues, not much is known about tissue expression of GPR173 in fish species. In goldfish, our study using RT-PCR revealed that PNXa, PNXb, and GPR173a were ubiquitously expressed in different tissues and brain areas. These patterns of PNX and GPR173 expression are highly comparable to zebrafish (27) and the wide range of tissue expression observed is also in line with the pleiotropic functions documented for PNX in different tissues (41, 42). Of note, the high level of GPR173 expression in the brain (especially in the hypothalamus) appears to be well conserved from fish to mammals, which can be correlated with the central actions of PNX including GnRH and Kiss regulation (20) and anxiolytic response for coping with anxiety (15). In our study, co-expression of PNXa, PNXb, and GPR173a were consistently detected in all the tissues and brain areas examined. Therefore, we do not exclude the possibility that PNXa/b can act as autocrine/paracrine signals and exert their effects via GPR173a expressed locally in the same tissue in our fish model.

In rat model, PNX is involved in food intake (10). Interestingly, a postprandial rise in plasma PNX has been reported in normal rats but this stimulatory effect is absent in obese rats (19). These findings imply that the PNX signal can be modified by food intake and energy balance in the body. This idea is congruent with the observation that patients with anorexia nervosa tend to have lower levels of plasma PNX, which can be partially normalized by therapeutic intervention to regain body weight (43). In fish models, the relationship between PNX expression and nutritional status is still controversial. In zebrafish, fasting has been reported to reduce PNX expression in tissues including the brain, gut, liver, gonads, and muscle (27). In spotted scat, similar fasting was found to upregulate PNX expression in the hypothalamus and this stimulatory effect could be reverted by refeeding (28). In our study with goldfish, food intake increased PNXa/b and GPR173a expression in the liver and brain areas including the telencephalon, hypothalamus, and optic tectum. A prolonged period of food deprivation, in contrast, was found to inhibit basal expression of the PNX/GPR173 system in the same tissue/brain areas. These results suggest that both peripheral and central expression of PNXa/b and GPR173a are under the control of food intake/nutrition status in our fish model. Our finding of PNX expression induced by feeding is consistent with the post-prandial rise in plasma PNX reported in the rats (19) and the PNX inhibition caused by food deprivation is also comparable to the corresponding response in zebrafish (27). Regarding the biological effects of PNX signals induced by food intake, IP and ICV injections of PNX20a and PNX20b were shown to upregulate surface foraging and food consumption in goldfish with parallel rises in locomotion distance, swimming velocity, duration of rapid swimming, and the time spent in upper half of the water close to the surface. Our findings of feeding induction by PNX20a/b in goldfish are similar to the reports in rats (10) and chickens (26) but opposite to the anorexic effect in zebrafish (27). Apparently, PNX regulation of food intake in fish models is species-specific. In rats, PNX14 not only can stimulate food intake but also induce drinking and locomotion activity, probably via activation of nesfatin-1 neurons in different brain areas (9). In goldfish, food intake induced by PNX20a/b was a direct consequence of the rise in surface foraging. The parallel changes in various parameters for body motility and spatial preference of locomotion also indicate that the swimming activity close to the water surface was enhanced during surface foraging, which is expected to be beneficial for food searching. Since the results of IP *vs* ICV injection of PNX20a/b on food intake, foraging behavior, and surface motility were similar, it would be logical to conclude that PNX20 can act within the brain to trigger parallel changes in body motility and surface foraging. In our study, (i) food consumption increased PNXa/b and GPR173a expression in brain areas involved in appetite control, and (ii) PNX20a/b induced foraging activity and food intake via central actions within the brain. Our findings raise the possibility that PNX may act as a feedforward signal with an orexigenic effect in the brain induced by food intake to maintain/prolong the feeding phase during a meal in goldfish. Of note, besides the central responses, food intake also induced PNXa/b and GPR173a expression in the liver. Therefore, the peripheral actions of PNX on feeding control cannot be excluded in our fish model.

In mammals, the feeding circuitry in the hypothalamus together with the solitary tract in the brainstem are known to play a key role in appetite control (44) and dysregulation in the hypothalamus caused by diseases/injury can lead to clinical obesity (45). Although the brain areas forming the feeding circuitry in fish are not fully identical to those in mammals due to different patterns of brain development (e.g., evagination pattern of forebrain development in mammals *vs* eversion pattern found in fish species), the neuroendocrine components for central regulation of food intake are well-conserved in fish models (46). In goldfish, previous studies based on lesioning of different brain areas have confirmed that the telencephalon, hypothalamus, and optic tectum are the key areas in the brain for appetite control and feeding behaviors (34, 47). Furthermore, the orexigenic factors, including NPY (48), AgRP (49), orexin (50), apelin (51), and ghrelin (52) and the anorexigenic factors, including αMSH (53), CART (54), CCK (55), CRH (56), MCH (57), leptin (58), and AdipoQ (36), are all expressed in these brain areas and involved in central control of food intake. For the mechanisms underlying feeding regulation by PNX, except for the two reports published (with one in chicken and the other in zebrafish), not much is known about the downstream signals mediating the central actions of PNX. In chickens, food consumption induced by ICV injection of PNX14 could be negated by the antagonist for the NPY receptor, suggesting the possible involvement of NPY in PNX action (26). In zebrafish, however, IP injection of PNX20 was found to reduce food intake and this feeding inhibition was probably mediated by increasing the CART signal with a concurrent drop in ghrelin expression in the hypothalamus (27). In our study with goldfish, IP and ICV injections of PNX20a/b were both effective in upregulating the expression of orexigenic factors (NPY, AgRP, orexin, and apelin) and their receptors (NPY1R, GHSR_1A1_, and GHSR_1A2_) in the telencephalon, hypothalamus, and/or optic tectum. Meanwhile, parallel drops in anorexigenic factors (POMC, CCK, CRH, and MCH) and their receptors (LepR, AdipoR_1_, and AdipoR_2_) were also observed in these brain areas. These findings suggest that PNX-induced food intake in goldfish was mediated by (i) differential modulation of the signals for orexigenic factors *vs* those of anorexigenic factors expressed within the brain, and (ii) readjusting the sensitivity for orexigenic/anorexigenic factors by regulating their receptor expression in brain areas involved in feeding control.

Although the results based on IP and ICV injections of PNX20a/b are highly comparable, variations in the kinetics and brain areas for target gene expression did occur in our study. These variabilities are suspected to be caused by the peripheral actions of PNX. In goldfish, IP injection of PNX20a/b reduced leptin 2, AdipoQ, SLβ, and IGF-I expression in the liver. Leptin (58) and AdipoQ (36) have been confirmed to be the anorexic factors in goldfish while SLβ is known to inhibit food intake in fish models, e.g., gilthead seabream (59). Although the role of IGF-I in appetite control has not been studied in fish species, IGF-I is well-documented to attenuate food intake via central actions in mammals (60, 61) and birds (62). In broiler chicks, the feeding inhibition by IGF-I is mediated by increasing the POMC signal in the hypothalamus (63). In goldfish, IP injections of leptin (58) and AdipoQ (36) are known to induce differential expression of NPY, AgRP, POMC, CART, and MCH at the hypothalamic level. It is likely that the feeding regulators from the liver induced by PNX may exert a secondary effect acting in the brain to modify the central expression of orexigenic/anorexigenic factors and their receptors. In the brain areas examined in our study, despite the downregulation of “anorexigenic receptors” observed after PNX20a/b treatment (e.g., LepR and AdipoR1/R2), a transient rise in MC4R signals also occurred with a parallel increase of SPX expression in the liver. MC4R is the receptor for melanocortin with potent inhibition on feeding (64) while SPX is a satiety factor identified in goldfish (31). These findings raise the possibility that PNX, besides its feeding stimulation during a meal, can also enhance the sensitivity in the brain to melanocortin signals with a parallel induction of SPX input from the periphery, which may contribute to the satiation response for meal termination in our fish model.

**Figure 15:**
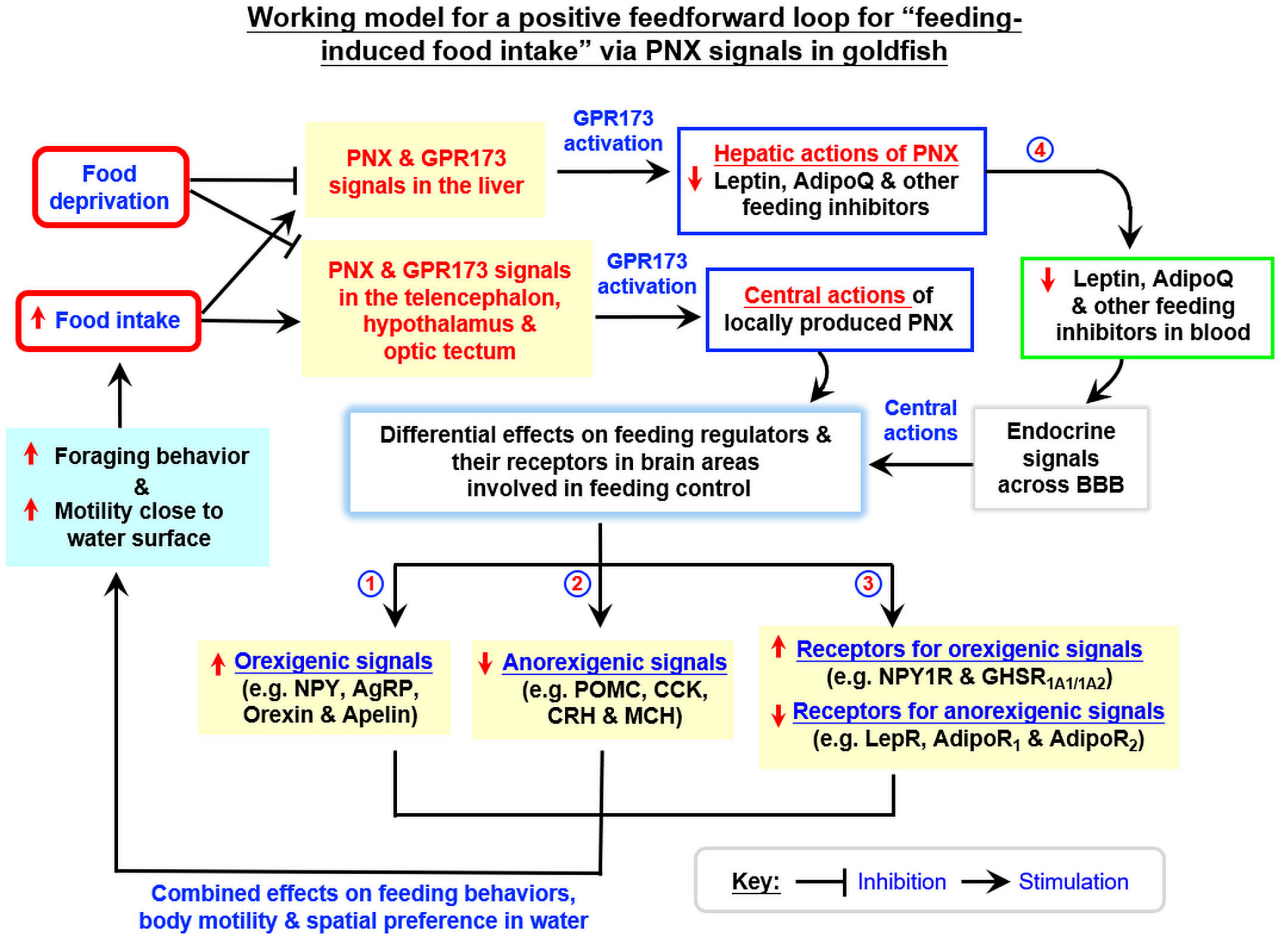
Working model for a positive feedforward loop for “feeding-induced food intake” via PNX signals in a fish model. In goldfish, food intake stimulates but food deprivation inhibits both hepatic and central expression of PNX and GPR173. PNX signals produced locally, presumably through GPR173 activation, can induce feeding by acting in the liver as well as in brain areas including the telencephalon, hypothalamus, and optic tectum. In these brain areas, PNX up-regulates orexigenic signals (NPY, AgRP, orexin, and apelin) with concurrent suppression of anorexigenic signals (POMC, CCK, CRH, and MCH). Meanwhile, the receptors for orexigenic factors (NPY1R and GHSR_1A1/1A2_) can also be up-regulated with parallel drops in the receptors for anorexigenic factors (LepR and AdipoR_1/2_). Besides the central actions, hepatic actions of PNX may form another functional component for feeding induction by PNX. In this case, PNX produced locally in the liver, probably via GPR173 activation, can reduce hepatic expression of anorexigenic factors (leptin, AdipoQ, IGF-I, and SLβ). The subsequent reduction of these anorexigenic signals in circulation presumably can reduce the inhibitory input for appetite control via the blood-brain barrier (BBB). The combined actions of the hepatic effects together with the central actions of PNX can induce foraging behavior with parallel rise in surface motility and lead to a positive feedforward loop of “feeding-induced food intake” in goldfish.

In summary, we have cloned and characterized the structural and evolutionary aspects of two forms of goldfish PNX, namely PNXa and PNXb. Tissue expression profiling also reveals that PNXa/b and their receptor GPR173a are ubiquitously expressed in peripheral tissues and different brain areas. Based on our *in vivo* studies, a working model has been proposed for the mechanisms underlying feeding regulation by PNX in goldfish (**Figure 15**). In this model, food intake increases but food deprivation inhibits PNXa, PNXb, and GPR173a expression in the liver and brain areas including the telencephalon, hypothalamus, and optic tectum. The upregulation of PNX signals in the three brain areas, presumably via GPR173 activation, can (i) induce local expression of orexigenic factors (e.g., NPY, AgRP, orexin, and apelin), (ii) reduce local signals of anorexigenic factors (e.g., POMC, CCK, CRH, and MCH), and (iii) differentially regulate the sensitivity to orexigenic (by increasing the receptors for NPY and ghrelin) and anorexigenic signals (by decreasing the receptors for leptin and AdipoQ). These central effects of PNX signals can then activate foraging behavior and body motility close to the water surface and lead to a rise in food intake. Of note, food intake can also elevate PNX and GPR173 expression in the liver. Possibly through GPR173 activation, the PNX signals produced locally can inhibit the production of anorexigenic factors at the hepatic level (e.g., leptin and AdipoQ). The subsequent downregulation of their output into circulation further reduces the feeding inhibition within the brain and contributes to “feeding-induced food intake” via PNX signaling. Our findings for the first time unveil a previously unrecognized feedforward orexigenic component during a meal in a fish model, which may play a key role in maintaining/prolonging food intake during the feeding phase of a meal prior to the onset of the satiety signals for feeding termination.

## Data Availability

The datasets presented in this study can be found in online repositories. The names of the repository/repositories and accession number(s) can be found below: https://www.ncbi.nlm.nih.gov/genbank/, XM_026268684; https://www.ncbi.nlm.nih.gov/genbank/, XM_026216912.
