# Supplementary material for: Goldfish phoenixin: (I) structural characterization, tissue distribution, and novel function as a feedforward signal for feeding-induced food intake in fish model
Source: Front Endocrinol (Lausanne). 2025 Apr 29;16:1570716. doi: 10.3389/fendo.2025.1570716 (PMC12069048; doi:10.3389/fendo.2025.1570716)
Supplement: Supplementary file 1 [file DataSheet1.pdf]

Supplementary Table 1

Supplementary Table 1

Primers and PCR conditions for RT-PCR of target genes

Gene Target/ GenBank accession No.<br>Sequences of forward (F) & reverse primers (R)	PCR condition				Product size
	Denaturing	Annealing	Extension	Cycle	
PNXa (SMIM20a)/ XM_026268684<br>F: 5′-CGGTGGCCTTTTATCCGATCT-3′<br>R: 5′-AACAGGCTGCACATCTGCTTGA-3′	94℃<br>30 sec	59℃<br>30 sec	72℃<br>30 sec	x35	108 bp
PNXb (SMIM20b)/ XM_026216912<br>F: 5′-AGAGGCTTCATTGCAGCGGTT-3′<br>R: 5′-TTGGCTTGAAGGGATCAGACC-3′	94℃<br>30 sec	59℃<br>30 sec	72℃<br>30 sec	x35	165 bp
GPR173/ XM_026270294<br>F: 5′-CATCAGCCTGGTGGGTAACT-3′<br>R: 5′-TCGCTTGGAGTAAAAGCGGT-3′	94℃<br>30 sec	58℃<br>30 sec	72℃<br>30 sec	x35	301 bp
β-actin/ AB039726.2<br>F: 5′-CTGGTATTCGTGATGGACTCT-3′<br>R: 5′-AGCTCATAGCTCTTCTCCAG-3’	94℃<br>30 sec	56℃<br>30 sec	72℃<br>30 sec	X35	287 bp
