# Supplementary material for: Goldfish phoenixin: (I) structural characterization, tissue distribution, and novel function as a feedforward signal for feeding-induced food intake in fish model
Source: Front Endocrinol (Lausanne). 2025 Apr 29;16:1570716. doi: 10.3389/fendo.2025.1570716 (PMC12069048; doi:10.3389/fendo.2025.1570716)
Supplement: Supplementary file 2 [file DataSheet2.pdf]

Supplementary Table 2

Supplementary Table 2
Primers and PCR conditions for real-time PCR of target genes

**Gene Target/ GenBank accession No.**<br>**Sequences of forward (F) & reverse primers (R)**	**PCR condition & Signal capture**					**Product size, *Tm* value & PCR efficiency**
	**Denaturing**	**Annealing**	**Extension**	**Detection**	**Cycle**	
PNXa (SMIM20a)/ XM_026268684						108 bp
F: 5′-CGGTGGCCTTTTATCCGATCT-3′	94℃	59℃	72℃	80℃	X35	Tm = 92 ℃
R: 5′-AACAGGCTGCACATCTGCTTGA-3′	30 sec	30 sec	30 sec	20 sec		(103.5%)
PNXb (SMIM20b)/ XM_026216912						165 bp
F: 5′-AGAGGCTTCATTGCAGCGGTT-3′	94℃	59℃	72℃	82℃	X35	Tm = 88 ℃
R: 5′-TTGGCTTGAAGGGATCAGACC-3′	30 sec	30 sec	30 sec	20 sec		(102.0%)
GPR173/ XM_026270294						301 bp
F: 5′-CATCAGCCTGGTGGGTAACT-3′	94℃	58℃	72℃	85℃	X35	Tm = 90 ℃
R: 5′-TCGCTTGGAGTAAAAGCGGT-3′	30 sec	30 sec	30 sec	20 sec		(104.1%)
NPY/ M87297						234 bp
F: 5′-GTAGTGTTGCGGGTAGCGA-3′	94℃	64℃	72℃	88℃	X35	Tm = 92 ℃
R: 5′-CAGACACCCCGACCCAAG-3′	30 sec	30 sec	30 sec	20 sec		(94.5%)
AgRP/ AJ555492						230 bp
F: 5′-TGGCATCACATCCAAACCT-3′	94℃	64℃	72℃	82℃	X35	Tm = 82 ℃
R: 5′-CAGGTGATGACCCAAGCAG-3′	30 sec	30 sec	30 sec	20 sec		(96.7%)
Orexin/ DQ923590						286 bp
F: 5′-GCAGAGCTGC-CATTGTTGACGTT-3′	94℃	64℃	72℃	79℃	X35	Tm = 82 ℃
R: 5′-AACCTTGTGATTACCTCAGGAGT-3′	30 sec	30 sec	30 sec	20 sec		(92.9%)
Apelin/ FJ755698						340 bp
F: 5′-GAGCATAGCAAAGAGCTGGA-3′	94℃	64℃	72℃	89℃	X35	Tm = 94 ℃
R: 5′-AACCTTGTGATTACCTCAGGAGT-3′	30 sec	30 sec	30 sec	20 sec		(94.7%)
POMC/ AJ431209						282 bp
F: 5′-AAGCGCTCCTACTCCATGGA-3′	94℃	60℃	72℃	83℃	X35	Tm=85 ℃
R: 5′-CTCGTCCCAGGACTTCATGAA-3′	30 sec	30 sec	30 sec	20 sec		(91.9%)
CART/ AF288810						171 bp
F: 5′-CCAAAGGACCCGAATCTGA-3′	94℃	64℃	72℃	72℃	X35	Tm = 90 ℃
R: 5′-TITGCCGATTCTTGACCCT-3′	30 sec	30 sec	30 sec	20 sec		(90.2%)
CCK/ CAU70865						197 bp
F: 5′-CCGCAGTCTCAGAAGATGGG-3′	94℃	64℃	72℃	87℃	X35	Tm = 91 ℃
R: 5′-GGAGGGGCTTCTGCGATA-3′	30 sec	30 sec	30 sec	20 sec		(100.1%)
MCH/ AM403730						272 bp
F: 5′-AGGCTTGAGCGAGAACTTGG-3′	94℃	64℃	72℃	86℃	X35	Tm = 91 ℃
R: 5′-CCCAGAAGACCTACACCTCCC-3′	30 sec	30 sec	30 sec	20 sec		(91.3%)
CRH/ AF098629						150 bp
F: 5′-GGCTCGGTAACAGAAACCAGAA-3′	94℃	60℃	72℃	82℃	X35	Tm = 88 ℃
R: 5′-AATTGCCATCCAAGCGACC-3′	30 sec	30 sec	30 sec	20 sec		(108.3%)
NPY1R/ XM_026204017.1						254 bp
F: 5′-CTCGGACAGCCATGTAGACC-3′	94℃	67℃	72℃	89℃	X35	Tm = 88 ℃
R: 5′-TCTGGCATTGTTCCACCTCC-3′	30 sec	30 sec	30 sec	20 sec		(91.6%)
GHSR1A1/ AB504275						380 bp
F: 5′-TAAATGTTGAGCAGCCCTTCGCGC-3′	94℃	65℃	72℃	85℃	X35	Tm = 92 ℃
R: 5′- GGGCATGCAGAGAAAAATAAA-3′	30 sec	30 sec	30 sec	20 sec		(93.1%)
GHSR1A2/ AB504276						373 bp
F: 5′-ACAGGTTGTATAAGTTGAGCG-3′	94℃	65℃	72℃	86℃	X35	Tm = 90 ℃
R: 5′-AGGCATGCAGAGAAAAATGAG-3′	30 sec	30 sec	30 sec	20 sec		(90.8%)
LepR/ EU911005						156 bp
F: 5′-CTGGCTTGAAGGTGAACGGAC-3′	94℃	65℃	72℃	78℃	X35	Tm = 87 ℃
R: 5′-TTGGGTGACAGTGCAGTAGTC-3′	30 sec	30 sec	30 sec	20 sec		(99.1%)
AdipoR1/ OQ447502						340 bp
F: 5′-ATGTCAGGCCAAATCAGGTCTG-3′	94℃	64℃	72℃	80℃	X35	Tm = 91 ℃
R: 5′-GATGACCATGTAGGAGGTAGTCATTG-3′	30 sec	30 sec	30 sec	20 sec		(94.8%)
AdipoR2/ OQ447504						320 bp
F: 5′-ATGAGTGCCAGCACAGATCACAG-3′	94℃	64℃	72℃	80℃		Tm = 88 ℃
R: 5′-CTGAAGATGCTCTTGAAACAAGCTCTG-3′	30 sec	30 sec	30 sec	20 sec	x35	(90.7%)
MC4R/ AJ534337						376 bp
F: 5′-TGGACCGCATCATTCATAC-3′	94℃	65℃	72℃	83℃	X35	Tm = 87 ℃
R: 5′-CAACAGTGAGCTGCAGATC-3′	30 sec	30 sec	30 sec	20 sec		(93.7%)

Supplementary Table 2 / Continued

Gene Target/ GenBank accession No. Sequences of forward (F) & reverse primers (R)	PCR condition & Signal capture					Product size, *Tm* value & PCR efficiency
	Denaturing	Annealing	Extension	Detection	Cycle	
Leptin 1/ FJ534535<br>F: 5′-TCCAAAGCTCCTCATAGG-3′<br>R: 5′-TGGTGGGTGGCGTTTTCC-3′	94℃<br>30 sec	50℃<br>30 sec	72℃<br>30 sec	86℃<br>20 sec	X35	270 bp<br>Tm = 89 ℃<br>(104.4%)
Leptin 2/ FJ854572<br>F: 5′-TATCGTGGACACCCTAACTAC-3′<br>R: 5′-GGTCTAAAGCCAAGAACCCTAA-3′	94℃<br>30 sec	50℃<br>30 sec	72℃<br>30 sec	85℃<br>20 sec	X35	224 bp<br>Tm = 89 ℃<br>(107.1%)
AdipoQ/ ON087697<br>F: 5′-GTGCCAATCCGCTTCAACA-3′<br>R: 5′-CCTCATCTCCTGCCTCCAAA-3′	94℃<br>30 sec	62℃<br>30 sec	72℃<br>30 sec	83℃<br>20 sec	X35	250 bp<br>Tm = 88 ℃<br>(93.7%)
Ghrelin/ AF454389<br>F: 5′-GTAGTGTTGCGGGTAGCGA-3′<br>R: 5′-CAGACACCCCGACCCAAG-3′	94℃<br>30 sec	64℃<br>30 sec	72℃<br>30 sec	75℃<br>20 sec	X35	471 bp<br>Tm = 81 ℃<br>(98.2%)
Insulin/ KT071542.1<br>F: 5′-GATGCCCTCTACCTGGTCTG-3′<br>R: 5′-TCCTTATCAGCTCTGCGTGA-3′	94℃<br>30 sec	64℃<br>30 sec	72℃<br>30 sec	84℃<br>20 sec	X35	157 bp<br>Tm = 88 ℃<br>(91.4%)
IGF-I/ AF001005<br>F: 5′-TTCAAGTGTACCATGCGCTG-3′<br>R: 5′-ACCGTCTTGAATTAGGCCCA-3′	94℃<br>30 sec	62℃<br>30 sec	72℃<br>30 sec	85℃<br>20 sec	X35	202 bp<br>Tm = 82 ℃<br>(98.4%)
SLα/ EU580712<br>F: 5′-ATATGTTTGTCCCGTACCCTCT-3′<br>R: 5′-TTTATCAGACACCCACTTGGTC-3′	94℃<br>30 sec	56℃<br>30 sec	72℃<br>30 sec	85℃<br>20 sec	X35	348 bp<br>Tm = 89 ℃<br>(106.5%)
SLβ/ CAU72940<br>F: 5′-AGGGACCATGTGTTCTCCTAAA-3′<br>R: 5′-AGAACCAGTATACCCTGCTCCA-3′	94℃<br>30 sec	62℃<br>30 sec	72℃<br>30 sec	83℃<br>20 sec	X35	238 bp<br>Tm = 86 ℃<br>(102.0%)
18S RNA/ HQ615531<br>F: 5′-AGCAACTTTAGTATACGCTATTGGAG-3′<br>R: 5′-CCTGAGAAACGGCTACCACATCC-3′	94℃<br>30 sec	56℃<br>30 sec	72℃<br>30 sec	87℃<br>20 sec	X35	285 bp<br>Tm = 88 ℃<br>(94.7%)
