# Supplementary material for: Goldfish phoenixin: (I) structural characterization, tissue distribution, and novel function as a feedforward signal for feeding-induced food intake in fish model
Source: Front Endocrinol (Lausanne). 2025 Apr 29;16:1570716. doi: 10.3389/fendo.2025.1570716 (PMC12069048; doi:10.3389/fendo.2025.1570716)
Supplement: Supplementary file 3 [file DataSheet3.pdf]

Supplementary Fig.1

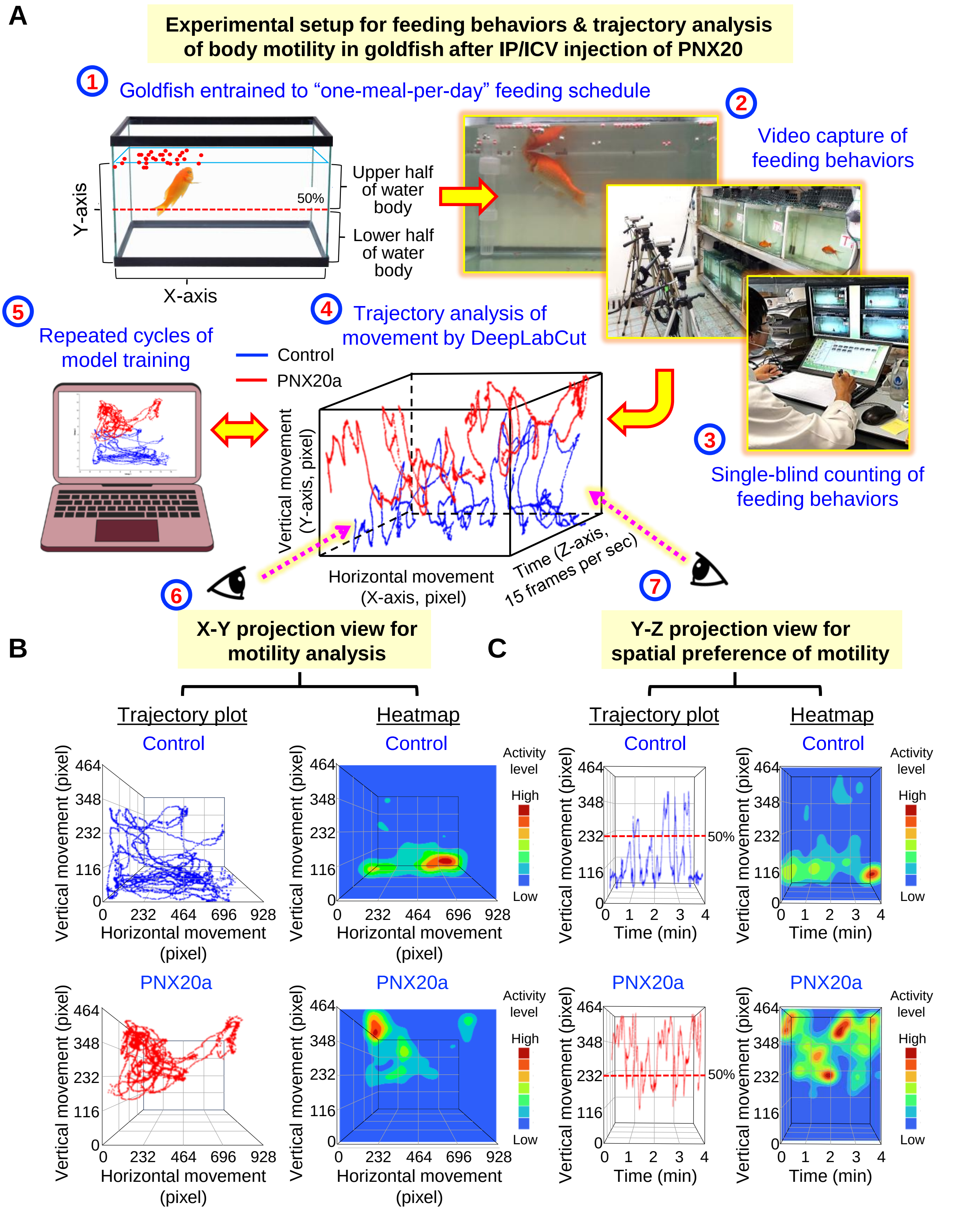

Supplementary Fig.1 Experimental setup and data analysis of goldfish feeding and body motility. Goldfish were subjected to IP/ICV injection of PNX20 followed by video recording of their feeding behaviors and body motility. Different types of feeding behaviors were scored manually and trajectory analysis of body motility was conducted with DeepLabCut (A). After model training, coordinate data of body movement were extracted for construction of trajectory plots and heat maps for (B) motility analysis (based on X-Y projection view) and (C) spatial preference of movement (based on Y-Z projection view). Data presented for model training were based on the 4-min clips of videos taken from goldfish with/without IP injection of PNX20a.
