# Supplementary material for: Goldfish phoenixin: (I) structural characterization, tissue distribution, and novel function as a feedforward signal for feeding-induced food intake in fish model
Source: Front Endocrinol (Lausanne). 2025 Apr 29;16:1570716. doi: 10.3389/fendo.2025.1570716 (PMC12069048; doi:10.3389/fendo.2025.1570716)
Supplement: Supplementary file 4 [file DataSheet4.pdf]

Supplementary Fig.2

A

Goldfish PNXa/SMIM20a

```
1                  ttttaatattccccccggaagagcattgaagagttg
37   attcctgtcctcggttgaggtgaatcaatcttttaaataatcataatc
85   ATGTCTGCTAACAGGAGGATAACGCTCATATTTGGAGGCTTCGTTGCA
      M  S  A  N  R  R  I  T  L  I  F  G  G  F  V  A   16
133  GCAGTCGCGGTGGCCTTTTATCCGATCTTCTTCCATCCTCTCACTCAC
      A  V  A  V  A  F  Y  P  I  F  F  H  P  L  T  H   32
181  ACTGAAGACTACAAACAGATGCAGAAGGTAAATCGAGCCGGAATCAAT
      T  E  D  Y  K  Q  M  Q  K  V  N  R  A  G  I  N   42
229  CAAGCAGATGTGCAGCCTGCTGGTGTGAAGATCTGGTCTGATCCATTC
      Q  A  D  V  Q  P  A  G  V  K  I  W  S  D  P  F   64
277  AAGCCAAAATCATGAttccagtgtcccgaggaaaattgagtgaataaa
      K  P  K  S  *
325  caggaggattgatgtgccacgctctgaggatcgagtttttgagacaaa
373  cacattaattctttgcaaccatgcatcaaatgagacttttaggatgta
421  tggatcttaatgtgtgtgtagatttgtgtgggaatgtgattgaaact
469  agaaagaacgaattactaaaatgagttcaaataaaataaaaatgtaaa
517  ctttgttgctttgttagtcagcacttcaaaatattatgttcatgaatc
565  acttttaatatttaagcttgtgtattaatggagtaacatattttgctg
613  tttgcattctgattctgaataaaaatatacatcttgaa
```

B

Goldfish PNXb/SMIM20b

```
1                            aaataaatgaccctcaccccggaaatgc
29   ATGAAAGAATTGATTTCTGTCCTCAGATGTGGTGAATCTATCAAAATC
      M  K  E  L  I  S  V  L  R  C  G  E  S  I  K  I   16
77   ATGTCTGCTAACAGGAGGATAACGCTCATATTCGGAGGCTTCATTGCA
      M  S  A  N  R  R  I  T  L  I  F  G  G  F  I  A   32
125  GCGGTTGCGGTGGCTTTTTATCCCATATTTTTCCATCCTCTCACTCAC
      A  V  A  V  A  F  Y  P  I  F  F  H  P  L  T  H   48
173  ACTGAAGACTACAAGCAGATCCAGAAGACAAACCGAGCCGGAGTCAAT
      T  E  D  Y  K  Q  I  Q  K  T  N  R  A  G  V  N   64
221  CAAGCAGATGTGCAGCCTGCTGGTCTGAAGATCTGGTCTGATCCCTTC
      Q  A  D  V  Q  P  A  G  L  K  I  W  S  D  P  F   80
269  AAGCCAAAATCATGAttcccgtgtgtggattaaagtttagtgaatgaa
      K  P  K  S  *                                    84
317  catgaggactgatgtgatgtcactcactgaggatggagtatttgagat
365  aaacactactaccatgcattaaatgagactttatggatgtatgaatct
413  tctgagtatgtatattttctgtgggaattaaaatgattgaacctagaa
461  tgacccaataaataataaaactttgttagtcagcatttaaaatatta
509  tgttcatgaatcactttgaatagttgaactaatgttggtgtattaatg
557  gattcctttatttgtattctaaataaaatacgcatattgaactgtt
```

Supplementary Fig.2 Nucleotide and protein sequences of goldfish PNX/SMIM20. Two forms of goldfish PNX/SMIM20 have been cloned, namely (A) PNXa/SMIM20a (GenBank Accession No. XM_026268684) and (B) PNXb/SMIM20b (GenBank Accession No. XM_02621691284). The ORFs of PNXa/SMIM20a and PNXb/SMIM20b are shown in upper cases (with deduced protein sequences underneath) and the 5'/3' UTRs are shown in lower cases. The protein sequence of PNX/SMIM20 is composed of an N-terminal followed by a transmembrane domain (shaded in grey) linking with the C-terminal (with the mature peptide PNX20 in red) via an 18 a.a. linker. The region covering PNX14, a truncated form of PNX20 with bioactivity, is underlined in red. The stop codon at the end of the respective ORF is marked by an asterisk (*) while the polyadenylation signals in the 3' UTRs are underlined in black.
