# Supplementary material for: Goldfish phoenixin: (I) structural characterization, tissue distribution, and novel function as a feedforward signal for feeding-induced food intake in fish model
Source: Front Endocrinol (Lausanne). 2025 Apr 29;16:1570716. doi: 10.3389/fendo.2025.1570716 (PMC12069048; doi:10.3389/fendo.2025.1570716)
Supplement: Supplementary file 5 [file DataSheet5.pdf]

Supplementary Fig.3

A

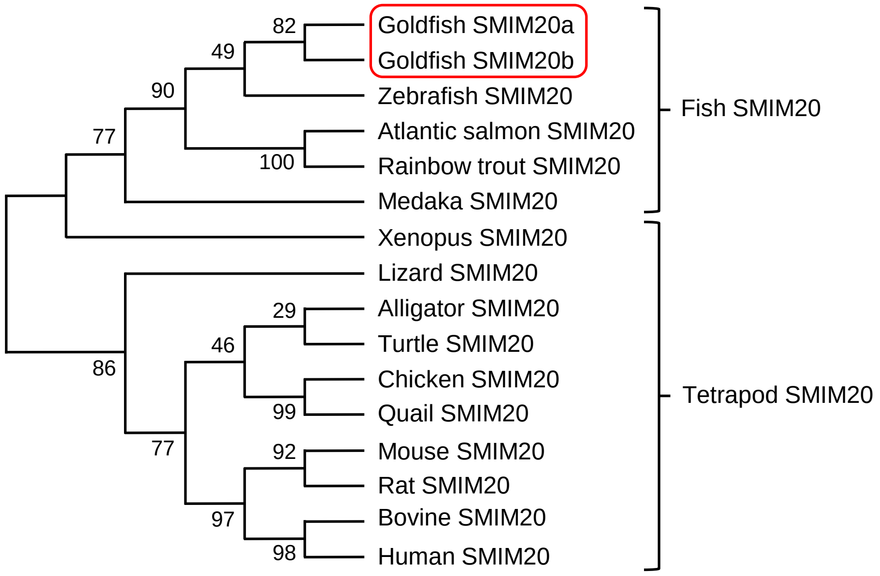

B

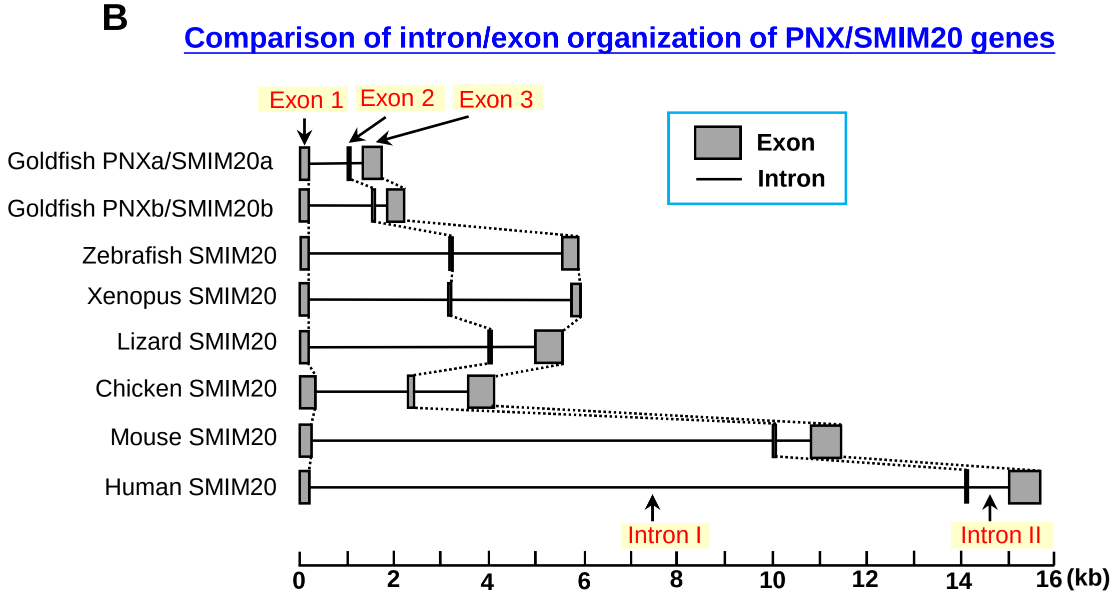

Supplementary Fig.3 Gene structure and phylogenetic analysis of PNX/SMIM20 in vertebrate species. (A) Phylogenetic analysis of goldfish PNXa/SMIM20a and PNXb/SMIM20b with their counterparts in species from different vertebrate classes using MEGA X with neighbour-joining method. The guide tree was constructed using PHYLIP 2.0 with the percentage of bootstrap values (based on 1,000 bootstraps) shown in individual nodes. (B) Comparison of intron/exon organization in goldfish PNXa/SMIM20a and PNXb/SMIM20b with the SMIM20 genes from other species. The gene sequences of SMIM20 in representative species from fish to mammals were downloaded from NCBI genome database and analyzed with Splicing Finder 2.4 to define the intron/ exon junctions for subsequent comparison of PNX/SMIM20 gene structure.
