# Supplementary material for: Goldfish phoenixin: (I) structural characterization, tissue distribution, and novel function as a feedforward signal for feeding-induced food intake in fish model
Source: Front Endocrinol (Lausanne). 2025 Apr 29;16:1570716. doi: 10.3389/fendo.2025.1570716 (PMC12069048; doi:10.3389/fendo.2025.1570716)
Supplement: Supplementary file 6 [file DataSheet6.pdf]

Supplementary Fig.4

Goldfish GPR173a

```
1     ATGGCAAACGGAAACGCAAGCAGCGATGGGCCTGGGAACCCCTTGGCGGCCGTGGTGTCC
       M  A  N  G  N  A  S  S  D  G  P  G  N  P  L  A  A  V  V  S   20
61    ACGACAGGTGGCGTGATGGGTGGAGCACCTTCCTCGGCCGTATCTACCTATGTGAAACTT
       T  T  G  G  V  M  G  G  A  P  S  S  A  V  S  T  Y  V  K  L   40
121   GTCCTTCTGGGCTTGATTATCTGCATCAGCCTGGTGGGTAACTTAGTGGTATCCCTGTTA
       V  L  L  G  L  I  I  C  I  S  L  V  G  N  L  V  V  S  L  L   60
181   GTGTTACGGGACAGAGCCCTACACAAGGCACCCTACTACTTTCTTCTGGACCTCTGCCTC
       V  L  R  D  R  A  L  H  K  A  P  Y  Y  F  L  L  D  L  C  L   80
241   GCCGACACCATACGCTCGGCAGTCTGCTTTCCCTTTGTCCTGGTGTCTATTAAGAACGGC
       A  D  T  I  R  S  A  V  C  F  P  F  V  L  V  S  I  K  N  G  100
301   TCAGCCTGGACTTACAGCGTGCTCAGCTGCAAGGTGGTGGCCTTCATGGCCGTCCTTTTT
       S  A  W  T  Y  S  V  L  S  C  K  V  V  A  F  M  A  V  L  F  120
361   TGCTTCCATGCTGCCTTCATGCTGTTCTGCATTAGCGTCACACGCTACATGGCCATCGCC
       C  F  H  A  A  F  M  L  F  C  I  S  V  T  R  Y  M  A  I  A  140
421   CACCACCGCTTTTACTCCAAGCGAATGACCTTCTGGACCTGCATTGCGGTGGTGTGCATG
       H  H  R  F  Y  S  K  R  M  T  F  W  T  C  I  A  V  V  C  M  160
481   GTCTGGACGCTGTCAGTCGCCATGGCCTTCCCACCTGTCTTTGACGTTGGCACCTACAAG
       V  W  T  L  S  V  A  M  A  F  P  P  V  F  D  V  G  T  Y  K  180
541   TTCATCCGCGAGGAGGACCAGTGCATCTTTGAGCATCGCTACTTCAAGGCCAATGACACG
       F  I  R  E  E  D  Q  C  I  F  E  H  R  Y  F  K  A  N  D  T  200
601   CTAGGCTTCATGCTGATGCTGGCTGTGCTAATCCTGGCTACTCACGTGGTCTACATGAAG
       L  G  F  M  L  M  L  A  V  L  I  L  A  T  H  V  V  Y  M  K  220
661   CTCCTGCTGTTCGAATATAAGCACCGAAAGATGAAGCCAGTCCAGATGGTTCCAGCCATC
       L  L  L  F  E  Y  K  H  R  K  M  K  P  V  Q  M  V  P  A  I  240
721   AGCCAAAACTGGACCTTTCACGGACCTGGAGCCACAGGCCAGGCAGCCGCAAACTGGATA
       S  Q  N  W  T  F  H  G  P  G  A  T  G  Q  A  A  A  N  W  I  260
781   GCAGGGTTCGGCCGTGGCCCAATGCCCCCCACTTTATTGGGCATCAGGCAGAACTTGCAC
       A  G  F  G  R  G  P  M  P  P  T  L  L  G  I  R  Q  N  L  H  280
841   AACCAGAACAGACGCCTGCTAGGCATGGAGGAGTTTAAGGCCGAGAAGCAGCTTGGTAGG
       N  Q  N  R  R  L  L  G  M  E  E  F  K  A  E  K  Q  L  G  R  300
901   ATGTTCTATGTCATCACCTTGTTTTTCTGGTGCTCTGGTCCCCGTACATTGTGGCCTGT
       M  F  Y  V  I  T  L  F  F  L  V  L  W  S  P  Y  I  V  A  C  320
961   TACTGGCGGGTCTTTGTGAAGGCGTGCACCATCCCGCACCGGTATCTTTCCACCACCGTG
       Y  W  R  V  F  V  K  A  C  T  I  P  H  R  Y  L  S  T  T  V  340
1021  TGGATGAGTTTTGCCCAAGCTGGCGTGAACCCCATCATCTGCTTTTTCCTCAACAAGGAC
       W  M  S  F  A  Q  A  G  V  N  P  I  I  C  F  F  L  N  K  D  360
1081  CTGAAGAAGGGCCTGCTGACCCACCTGCCTCCCTGCTGTAGAACTCCACCTCAACTGCCC
       L  K  K  G  L  L  T  H  L  P  P  C  C  R  T  P  P  Q  L  P  380
1141  CGTGAGCCTTACTGTGTCATGTGA
       R  E  P  Y  C  V  M  *                                       387
```

Supplementary Fig.4 Nucleotide and protein sequences of goldfish GPR173a. GPR173a (GenBank Accession No. XM_026270294) has been cloned in goldfish using 3'/5' RACE and the ORF obtained (1164 bp) is shown in upper cases with the deduced a.a. sequence underneath. In the protein sequence of GPR173a, the N-linked glycosylation site in the N-terminal is labelled in red and the regions for the seven transmembrane domains characteristic of the G-protein coupled receptors are shaded with a grey background. The stop codon at the end of the ORF is marked by an asterisk (*).
