# Supplementary material for: Goldfish phoenixin: (I) structural characterization, tissue distribution, and novel function as a feedforward signal for feeding-induced food intake in fish model
Source: Front Endocrinol (Lausanne). 2025 Apr 29;16:1570716. doi: 10.3389/fendo.2025.1570716 (PMC12069048; doi:10.3389/fendo.2025.1570716)
Supplement: Supplementary file 7 [file DataSheet7.pdf]

Supplementary Fig.5

Sequence alignment of GPR173 in different vertebrates

```
Goldfish GPR173a            .......MANGNASSDGPGNPLAAVVSTTG................GVMGGAPSSAVSTYVKLVLLGLII
Zebrafish GPR173a           .......MANGNASSDGPGNPLAAVVSTTG................GVMGGAPSSAVSTYVKLVLLGLII
Spotted scat GPR173a        MGGGAFGMANGNESSEGLAGPMAAVVATAA................GLVAESPSSAVSTYIKLVLLGLII
Medaka GPR173a              .......MANGSESSEVLVEPMAAVVATAA................GMLPESSSSTVSTYIKLVLLGLII
Atlantic salmon GPR173a1    .......MANGNETSEGLGGPLAAVVATTG................GMAVGGPSSAVSTYIKLVLLGLII
Rainbow trout GPR173a1      .......MANGNETSEGLGGPLAAVVATTG................GMAVGGPSSAVSTYIKLVLLGLII
Atlantic salmon GPR173a2    .......MANGNESSEGLGGPMAAVVATTG................GMAVGGPSSAVSTYIKLVLLGLII
Rainbow trout GPR173a2      -------MANGNESSEGLGGPMAAVVATTG................GMAVGGPSSVVSTYIKLVLLGLII
Xenopus GPR173              -------MANGS-ETEELSGSL.............................SQSTVISTHLKLVLLGFII
Lizard GPR173               -------MASAN-ETEEAHG...............................SAPHAASTYAKLLLLGLII
Bovine GPR173               -------MANTTGEPEEVSGAL-----------------------------SPPSAV-AYVKLVLLGLIM
Mouse GPR173                -------MANTTGEPEEVSGAL-----------------------------SLPSAS-AYVKLVLLGLIM
Human GPR173                -------MANTTGEPEEVSGAL-----------------------------SPPSAS-AYVKLVLLGLIM
Spotted scat GPR173b        -------MANQSFAIDGPGSLLAVLASQSGLARSSSSSSSS-SDSSSNSGGISATDVSAYFKLVFLGLII
Mummichog GPR173b           -------MANQSFAIDGPGSLLAVLASQSGLAKGGSSSGSS-GDGSGSNGGITATDVSAYFKLVFLGLII
Guppy GPR173b               -------MSNQSFAIDGPGSLLAVLASQSGQAKGGSSSSSSDGNSDGSSGGLSATDVSAYFKLVFLGLII
```

```
                            TMD1                            TMD2                         *
Goldfish GPR173a            CISLVGNLVVSLLVLRDRALHKAPYYFLLDLCLADTIRSAVCFPFVLVSIKNGSAWTYSVLSCKVVAFMA
Zebrafish GPR173a           CISLVGNLVVSLLVLRDRALHKAPYYFLLDLCLADTIRSAVCFPFVLVSIKNGSAWTYSVLSCKVVAFMA
Spotted scat GPR173a        CVSLVGNLVVSLLVLRDRALHKAPYYFLLDLCLADTIRSAICFPFVLVSIKNGSAWTYSVLSCKVVAFMA
Medaka GPR173a              FISLMGNLMVSLLVLRNRMLHKAPYYFLLDLCLADTIRSAICFPFVLVSIKNGSAWTYSVLSCKVVAFMA
Atlantic salmon GPR173a1    CISLVGNLVVSLLVLRDRALHKAPYYFLLDLCLADTIRSAVCFPFVLVSIKNGSAWTYSVLSCKVVAFMA
Rainbow trout GPR173a1      CISLVGNLVVSLLVLRDRALHKAPYYFLLDLCLADTIRSAVCFPFVLVSIKNGSAWTYSVLSCKVVAFMA
Atlantic salmon GPR173a2    CISLVGNLVVSLLVLRDRALHKAPYYFLLDLCLADTIRSAVCFPFVLVSIKNGSAWTYSVLSCKVVAFMA
Rainbow trout GPR173a2      CISLVGNLVVSLLVLRDRALHKAPYYFLLDLCLADTIRSAVCFPFVLVSIKNGSAWTYSVLSCKVVAFMA
Xenopus GPR173              CASLAGNLLVSLLVLKDRSLHKAPYYFLLDLCLADAVRSSACFPFVLLSIRNGSAWAYSLLSCKVVAFMA
Lizard GPR173               CVSLAGNLSLSLLVLKERGLHKAPYYFLLDLCLADVIRSAVCFPFVLVSIRHGSAWTYSVLSCKIVAFMA
Bovine GPR173               CVSLAGNAILSLLVLKDRALHKAPYYFLLDLCLADGIRSAVCFPFVLASVRHGSSWTFSALSCKIVAFMA
Mouse GPR173                CVSLAGNAILSLLVLKERALHKAPYYFLLDLCLADGIRSAICFPFVLASVRHGSSWTFSALSCKIVAFMA
Human GPR173                CVSLAGNAILS-LVLKERALHKAPYYFLLDLCLADGIRSAVCFPFVLASVRHGSSWTFSALSCKIVAFMA
Spotted scat GPR173b        CVSLVGNLLVSLLVLRDRTLHKAPYFFLLDLCLADAVRSAACFPFVLVSVHNSSAWTYSALSCKVVAFMA
Mummichog GPR173b           CVSLVGNLLVSMLVLRDRTLHKAPYFFLLDLCLADAVRSAACFPFVLVSVHNSSAWTYSALSCKVVAFMA
Guppy GPR173b               CVSLVGNLLVSMLVLRDRTLHKAPYFFLLDLCLADAVRSAACFPFVLVSVHNSSDWPYSKLSCKVVAFMS
```

```
                              TMD3                                 TMD4
Goldfish GPR173a            VLFCFHAAFMLFCISVTRYMAIAHHRFYSKRMTFWTCIAVVCMVWTLSVAMAFPPVFDVGTYKFIREEDQ
Zebrafish GPR173a           VLFCFHAAFMLFCISVTRYMAIAHHRFYSKRMTFWTCVAVVCMVWTLSVAMAFPPVFDVGTYKFIREEDQ
Spotted scat GPR173a        VLFCFHAAFMLFCISVTRYMAIAHHRFYSKRMTFWTCVAVVCMVWTLSVAMAFPPVFDVGTYKFIREEDQ
Medaka GPR173a              VLFCFHAAFMLFCISVTRYMAIAHYRFYTKRMTFWTCVAVVCMVWTLSVAMAFPPVFDVGTYKFIREEDQ
Atlantic salmon GPR173a1    VLFCFHAAFMLFCISVTRYMAIAHHRFYSKRMTFWTCVAVVCMVWTLSVAMAFPPVFDVGTYKFIREEDQ
Rainbow trout GPR173a1      VLFCFHAAFMLFCISVTRYMAIAHHRFYSKRMTFWTCVAVVCMVWTLSVAMAFPPVFDVGTYKFIREEDQ
Atlantic salmon GPR173a2    VLFCFHAAFMLFCISVTRYMAIAHHRFYSKRMTFWTCVAVVCMVWTLSVAMAFPPVFDVGTYKFIREEDQ
Rainbow trout GPR173a2      VLFCFHAAFMLFCISVTRYMAIAHHRFYSKRMTFWTCVAVVCMVWTLSVAMAFPPVFDVGTYKFIREEDQ
Xenopus GPR173              VLFCFHASFMLFCISVTRYMAIAHHRFYSKRMTLWTCIAVICMVWTLSVAMAFPPVFDVGTYKFIREEDQ
Lizard GPR173               VLFCFHAAFLLFCISVTRYMAVAHHRFYAKRMTFWTCVAVICMVWTLSVAMAFPPVFDVGTYKFIREEDQ
Bovine GPR173               VLFCFHAAFMLFCISVTRYMAIAHHRFYAKRMTLWTCAAVICMAWTLSVAMAFPPVFDVGTYKFIREEDQ
Mouse GPR173                VLFCFHAAFMLFCISVTRYMAIAHHRFYAKRMTLWTCAAVICMAWTLSVAMAFPPVFDVGTYKFIREEDQ
Human GPR173                VLFCFHAAFMLFCISVTRYMAIAHHRFYAKRMTLWTCAAVICMAWTLSVAMAFPPVFDVGTYKFIREEDQ
Spotted scat GPR173b        VLFCFHAAFMLFCVAVTRYLAIAHHRFYAKRMTIWTCAAIICMVWTLAVAMAFPPVFDVGTYKFIRDEDQ
Mummichog GPR173b           VLFCFHAAFMLFCVAVTRYLAIAHHRFYAKRMTIWTCAAIICMVWTLAVAMAFPPVFDVGTYKFIRDEDQ
Guppy GPR173b               VLFCFHAAFMLFCVAVTRYLAIAHHRFYAKRMTIWTCAAIICMVWTLAVAMAFPPVFNVGTYEFIRDEDQ
```

```
                          *            TMD5
Goldfish GPR173a          CIFEHRYFKANDTLGFMLMLAVLILATHVVYMKLLLFEYKHRKMKPVQMVPAISQNWTFHGPGATGQAAA
Zebrafish GPR173a         CIFEHRYFKANDTLGFMLMLAVLILATHVVYMKLLLFEYKHRKMKPVQMVPAISQNWTFHGPGATGQAAA
Spotted scat GPR173a      CIFEHRYFKANDTLGFMLMLAVLILATHVVYMKLLLFEYKHRKMKPVQMVPAISQNWTFHGPGATGQAAA
Medaka GPR173a            CIFEHRYFKANDTLGFMLMLAVLILATHVVYMKLLLFEYKHRKMKPVQMVPAISQNWTFHGPGATGQAAA
Atlantic salmon GPR173a1  CIFEHRYFKANDTLGFMLMLAVLILATHVVYMKLLLFEYKHRKMKPVQMVPAISQNWTFHGPGATGQAAA
Rainbow trout GPR173a1    CIFEHRYFKANDTLGFMLMLAVLILATHVVYMKLLLFEYKHRKMKPVQMVPAISQNWTFHGPGATGQAAA
Atlantic salmon GPR173a2  CIFEHRYFKANDTLGFMLMLAVLILATHVVYMKLLLFEYKHRKMKPVQMVPAISQNWTFHGPGATGQAAA
Rainbow trout GPR173a2    CIFEHRYFKANDTLGFMLMLAVLILATHVVYMKLLLFEYKHRKMKPVQMVPAISQNWTFHGPGATGQAAA
Xenopus GPR173            CIFEHRYFKANDTLGFMLMLAVLIVATHIVYAKLLLFEYRHRKMKPVQMVPAISQNWTFHGPGATGQAAA
Lizard GPR173             CIFEHRYFKANDTLGFMLMLAVLIFATHVVYIKLLLFEYRHRKMKPVQMVPAISQNWTFHGPGATGQAAA
Bovine GPR173             CIFEHRYFKANDTLGFMLMLAVLMAATHAVYGKLLLFEYRHRKMKPVQMVPAISQNWTFHGPGATGQAAA
Mouse GPR173              CIFEHRYFKANDTLGFMLMLAVLMAATHAVYGKLLLFEYRHRKMKPVQMVPAISQNWTFHGPGATGQAAA
Human GPR173              CIFEHRYFKANDTLGFMLMLAVLMAATHAVYGKLLLFEYRHRKMKPVQMVPAISQNWTFHGPGATGQAAA
Spotted scat GPR173b      CIFEHRYLKTNDTLGFMLMLAVVVLATHGFYAKLLLFEYRHRKMKPVQLVPAISQNWTFHGPGATGQAAA
Mummichog GPR173b         CIFEHRYLKTNDTLGFMLMLAVVVLATHGFYAKLLLFEYRHRKMKPVQLVPAISQNWTFHGPGATGQAAA
Guppy GPR173b             CIFEHRHLKNNDTLGFMLMLAVVVLATHGFYAKLLLFEYRHRKMKPVQLVPAISQNWTFHGPGATGQAAA

                                                                              TMD6
Goldfish GPR173a          NWIAGFGRGPMPPTLLGIRQNLHNQNRRLLGMEEFKAEKQLGRMFYVITLFFLVLWSPYIVACYWRVFVK
Zebrafish GPR173a         NWIAGFGRGPMPPTLLGIRQNLHNQNRRLLGMEEFKAEKQLGRMFYVITLFFLVLWSPYIVACYWRVFVK
Spotted scat GPR173a      NWIAGFGRGPMPPTLLGIRQNLHNQNRRLLGMEEFKAEKQLGRMFYVITLLFLVLWSPYIVACYWRVFVK
Medaka GPR173a            NWIAGFGRGPMPPTLLGIRQNLHNQNRRLLGMEEFKVEKQLGRMFYVITLLFLLLWSPYIVACYWRVFVK
Atlantic salmon GPR173a1  NWIAGFGRGPMPPTLLGIRQNLHNQNRRLLGMEEFKAEKQLGRMFYVITLLFLVLWSPYIVACYWRVFVK
Rainbow trout GPR173a1    NWIAGFGRGPMPPTLLGIRQNLHNQNRRLLGMEEFKAEKQLGRMFYVITLLFLVLWSPYIVACYWRVFVK
Atlantic salmon GPR173a2  NWIAGFGRGPMPPTLLGIRQNLHNQNRRLLGMEEFKAEKQLGRMFYVITLLFLVLWSPYIVACYWRVFVK
Rainbow trout GPR173a2    NWIAGFGRGPMPPTLLGIRQNLHNQNRRLLGMEEFKAEKQLGRMFYVITLLFLVLWSPYIVACYWRVFVK
Xenopus GPR173            NWIAGFGRGPMPPTLLGIRQNTHTANRRLLGMDEFKSEKRLGRMFYGITLSFLVLWSPYIVACYWRVFVK
Lizard GPR173             NWIAGFGRGPMPPTLLGIRQNAHAANRRLLGMEEFKAEKRLGRMFYVITLLFLVLWSPYIVACYWRVFVK
Bovine GPR173             NWIAGFGRGPMPPTLLGIRQNGHAASRRLLGMDEVKGEKQLGRMFYAITLLFLLLWSPYIVACYWRVFVK
Mouse GPR173              NWIAGFGRGPMPPTLLGIRQNGHAASRRLLGMDEVKGEKQLGRMFYAITLLFLLLWSPYIVACYWRVFVK
Human GPR173              NWIAGFGRGPMPPTLLGIRQNGHAASRRLLGMDEVKGEKQLGRMFYAITLLFLLLWSPYIVACYWRVFVK
Spotted scat GPR173b      NWIAGFGRGPMPPTLLGIRQNLHNQHRRLLGMEEVRSERRLGRMFYTITLLFLVLWAPYIVACFWRVFVK
Mummichog GPR173b         NWIAGFGRGPMPPTLLGIRQNLHNQHRRLLGMEEVRSERRLGRMFYTITLLFLVLWAPYIVACFWRVFVK
Guppy GPR173b             NWIAGFGRGPMPPTLLGIRQNLHNQHRRLLGMEEVRSERRLGRMFYTITLLFLVLWAPYIVACFWRVFVQ

                                    TMD7                                          % homology
Goldfish GPR173a          ACTIPHRYLSTTVWMSFAQAGVNPIICFFLNKDLKKGLLTHLPPCCRTPPQLPR-EPYCVM   100.0
Zebrafish GPR173a         ACTIPHRYLSTTVWMSFAQAGVNPIICFFLNKDLKKGLLAHLPPCCRTPPQLPR-EPYCVM    99.7
Spotted scat GPR173a      ACTIPHQYLSTTVWMSFAQAGVNPIVCFFFNKDLKKGLLSHLPVCCRTKPHLPR-EPYCVM    94.9
Medaka GPR173a            ACTIPHRYLSTTVWMSFAQAGVNPIVCFFLNKDLKKGLLSHLPACCRTKPHLPR-EPYCVM    94.6
Atlantic salmon GPR173a1  ACTIPHRYLSTTVWMSFAQAGVNPIVCFFLNKDLKKGLLAHLPASCRTKPHLPR-EPYRVM    96.9
Rainbow trout GPR173a1    ACTIPHRYLSTTVWMSFAQAGVNPIVCFFLNKDLKKGLLAHLPASCRTKPHLPR-EPYRVM    96.9
Atlantic salmon GPR173a2  ACTIPHRYLSTTVWMSFAQAGVNPIVCFFLNKDLKKGLLAHLPACCRTKPHLPR-EPYFVM    97.6
Rainbow trout GPR173a2    ACTIPHRYLSTTVWMSFAQAGVNPIVCFFLNKDLKKGLLAHLPACCRTKPHLPR-EPYFVM    96.9
Xenopus GPR173            TCSIPHRYLSTAVWMTFTQAGVNPIMCFLLNKDLKSCLQLHIPCWRT-KALLPR-EPYCVM    88.1
Lizard GPR173             ACSIPHRYLSTAVWMSFAQAAVNPIVCFVLNKDLKKGLITHIPCWRT-EPELPR-EPYCVM    89.7
Bovine GPR173             ACAVPHRYLATAVWMSFAQAAVNPIVCFLLNKDLKKCLRTHAPCWGTGGAPAPR-EPYCVM    86.6
Mouse GPR173              ACAVPHRYLATAVWMSFAQAAVNPIVCFLLNKDLKKCLRTHAPCWGTGGAPAPR-EPYCVM    86.1
Human GPR173              ACAVPHRYLATAVWMSFAQAAVNPIVCFLLNKDLKKCLRTHAPCWGTGGAPAPR-EPYCVM    86.1
Spotted scat GPR173b      SCTIPHRYLSITVWMSFAQAGVNPIFCLLLNEDLRKVLRAHLPTYWRTKQHLPQDEAYCIM    85.0
Mummichog GPR173b         SCAIPHRYLSITVWMSFAQAGVNPIFCLLLNEDLRKVLRAHLPTYWRTKQHLPQDEAYCIM    85.0
Guppy GPR173b             SCTIPHRYLSITVWMSFAQAGVNPIFCLLLNEDLRKVLRAHLPTYWRTKQHLPQDEAYCIM    84.4
```

Supplementary Fig.5 Sequence alignment of goldfish GPR173a with the corresponding sequences in other species. The a.a. sequences of GPR173 in representative species from different vertebrate classes were downloaded from NCBI genome databases and aligned with that of goldfish GPR173a using Clustal-W algorithm. Conserved/homologous a.a. residues were boxed in grey and the seven transmembrane domains (TMD_{1-7}) identified were delineated with horizontal lines above the respective sequences. The two conserved cysteine residues for possible formation of an intramolecular disulfide bond bridging the first and second extracellular loops were marked by asterisks (*).
