# Supplementary material for: Goldfish phoenixin: (I) structural characterization, tissue distribution, and novel function as a feedforward signal for feeding-induced food intake in fish model
Source: Front Endocrinol (Lausanne). 2025 Apr 29;16:1570716. doi: 10.3389/fendo.2025.1570716 (PMC12069048; doi:10.3389/fendo.2025.1570716)
Supplement: Supplementary file 8 [file DataSheet8.pdf]

Supplementary Fig.6

2D Sequence analysis and domain comparison of GPR173

% Homology of subdomain structures in GPR173

	N-terminal	ICL_1	ECL_1	ICL_2	ECL_2	ICL_3	ECL_3	C-terminal
Goldfish GPR173a	100.0	100.0	100.0	100.0	100.0	100.0	100.0	100.0
Zebrafish GPR173a	100.0	100.0	100.0	100.0	100.0	100.0	100.0	96.7
Spotted scat GPR173a	68.1	100.0	100.0	100.0	100.0	100.0	100.0	90.0
Spotted scat GPR173b	38.7	90.9	84.2	100.0	82.9	98.7	100.0	66.7
Guppy GPR173b	41.0	90.9	73.7	100.0	92.9	98.7	100.0	66.7
Mummichog GPR173b	50.0	90.9	84.2	100.0	92.9	98.7	88.9	66.7
Xenopus GPR173	48.7	100.0	94.7	100.0	100.0	96.2	88.9	71.0
Lizard GPR173	35.9	90.9	100.0	100.0	100.0	96.2	100.0	90.0
Mouse GPR173	46.2	100.0	89.5	100.0	100.0	93.6	88.9	65.2
Human GPR173	48.7	100.0	89.5	100.0	100.0	93.6	88.9	65.6

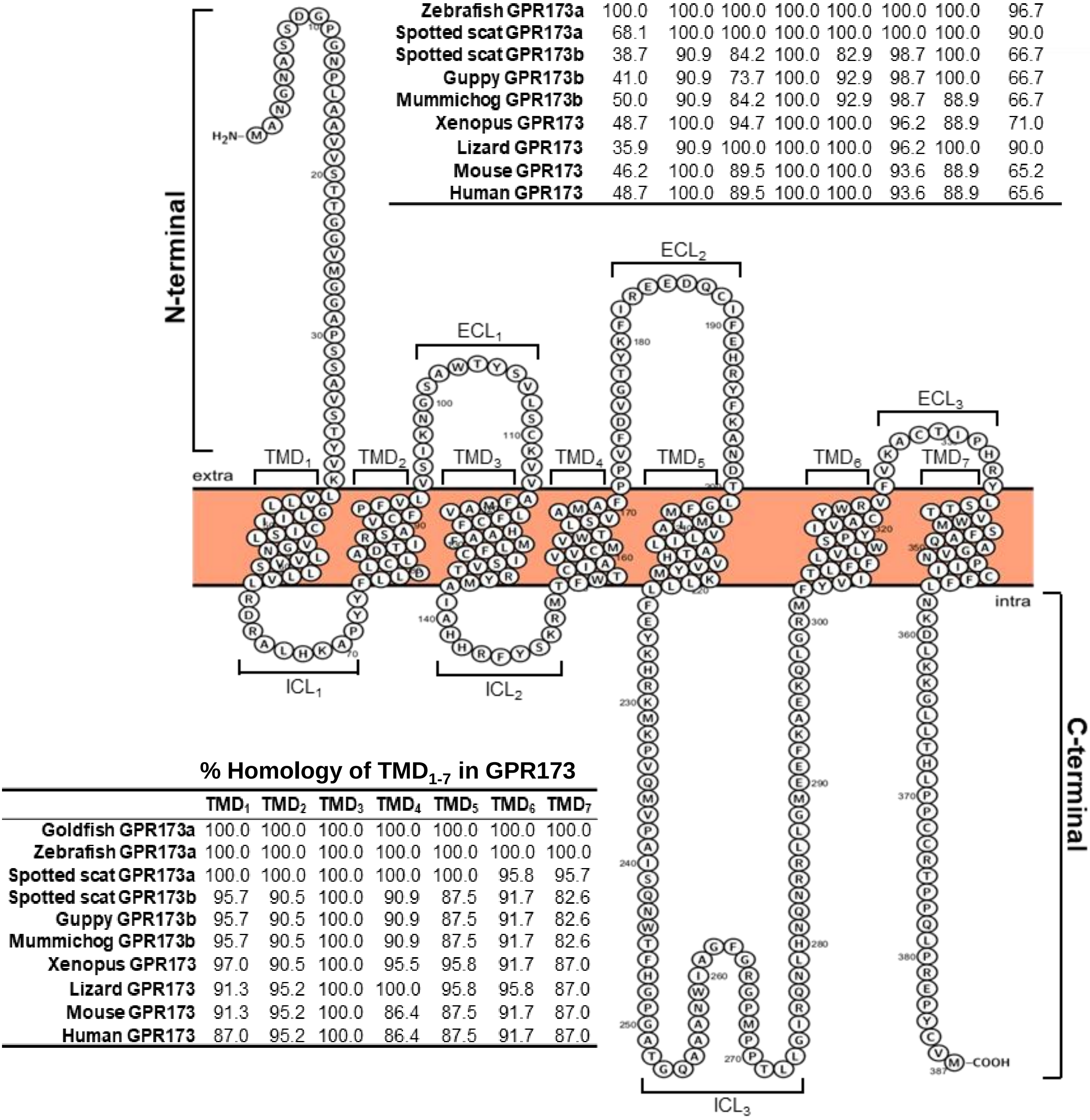

% Homology of TMD_{1-7} in GPR173

	TMD_1	TMD_2	TMD_3	TMD_4	TMD_5	TMD_6	TMD_7
Goldfish GPR173a	100.0	100.0	100.0	100.0	100.0	100.0	100.0
Zebrafish GPR173a	100.0	100.0	100.0	100.0	100.0	100.0	100.0
Spotted scat GPR173a	100.0	100.0	100.0	100.0	100.0	95.8	95.7
Spotted scat GPR173b	95.7	90.5	100.0	90.9	87.5	91.7	82.6
Guppy GPR173b	95.7	90.5	100.0	90.9	87.5	91.7	82.6
Mummichog GPR173b	95.7	90.5	100.0	90.9	87.5	91.7	82.6
Xenopus GPR173	97.0	90.5	100.0	95.5	95.8	91.7	87.0
Lizard GPR173	91.3	95.2	100.0	100.0	95.8	95.8	87.0
Mouse GPR173	91.3	95.2	100.0	86.4	87.5	91.7	87.0
Human GPR173	87.0	95.2	100.0	86.4	87.5	91.7	87.0

Supplementary Fig.6 2D Sequence analysis and subdomain delineation of goldfish GPR173a. Snake plot of goldfish GPR173a was constructed using Protter software. The 2D model deduced reveals that goldfish GPR173a is composed of an extracellular N-terminal followed by seven transmembrane domains (TMD_{1-7}) linked with the alternating intracellular (ICL_{1-3}) and extracellular loops (ECL_{1-3}) in between and intracellular C-terminal at the end. The two tables presented with the snake plot are the summary of percentage homology of the a.a. sequences for structural domains covering the different regions of GPR173 in species from fish to mammals.
