# Supplementary material for: Goldfish phoenixin: (I) structural characterization, tissue distribution, and novel function as a feedforward signal for feeding-induced food intake in fish model
Source: Front Endocrinol (Lausanne). 2025 Apr 29;16:1570716. doi: 10.3389/fendo.2025.1570716 (PMC12069048; doi:10.3389/fendo.2025.1570716)
Supplement: Supplementary file 9 [file DataSheet9.pdf]

Supplementary Fig.7

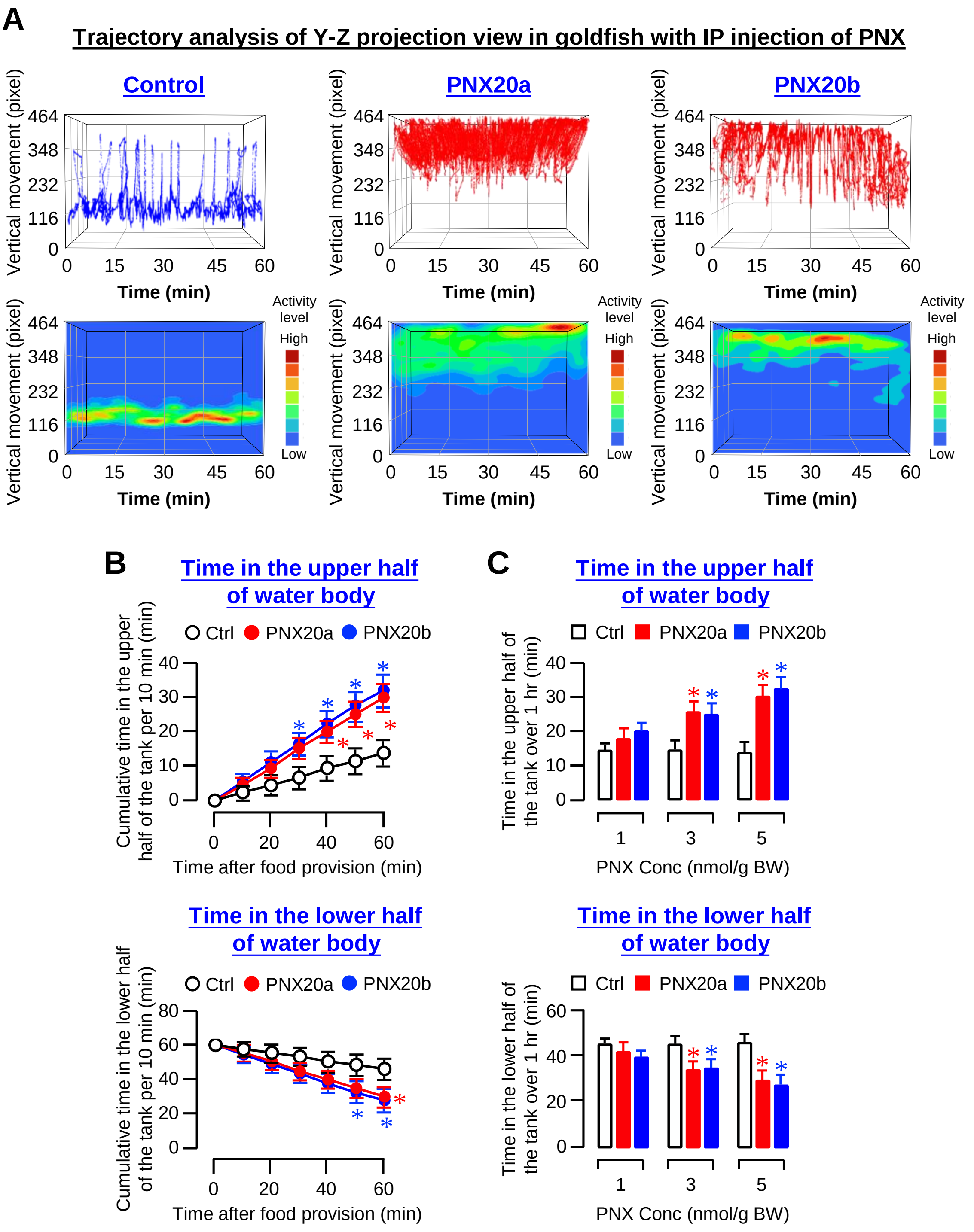

Supplementary Fig.7 Analysis of spatial preference of movement based on the Y-Z projection view of trajectory traces in goldfish with IP injection of PNX20a/b. Vertical movement associated with feeding in goldfish with IP injection (5 nmol/g BW) of PNX20a/b was recorded for the duration as indicated and with parallel injection of physiological saline as control. The videos obtained were analysed with DeepLabCut and coordinate data for vertical movement with respect to time were extracted for plotting the trajectory traces and heat maps for spatial preference of motility (A). For quantitative analysis of spatial preference, the cumulative time for goldfish staying in the upper half (upper panels) and lower half of water body (lower panels) were calculated based on the trajectory traces for (B) time course study with IP injection (5 nmol/g BW) of PNX20a/b up to 1 hr, and (C) dose-dependence study with IP injection of increasing levels (1-5 nmol/g BW) of PNX20a/b (with drug treatment for 1 hr). An asterisk (*) represents a significant difference ($p < 0.05$) compared to the respective control.
