# Supplementary material for: Goldfish phoenixin: (I) structural characterization, tissue distribution, and novel function as a feedforward signal for feeding-induced food intake in fish model
Source: Front Endocrinol (Lausanne). 2025 Apr 29;16:1570716. doi: 10.3389/fendo.2025.1570716 (PMC12069048; doi:10.3389/fendo.2025.1570716)
Supplement: Supplementary file 10 [file DataSheet10.pdf]

Supplementary Fig.8

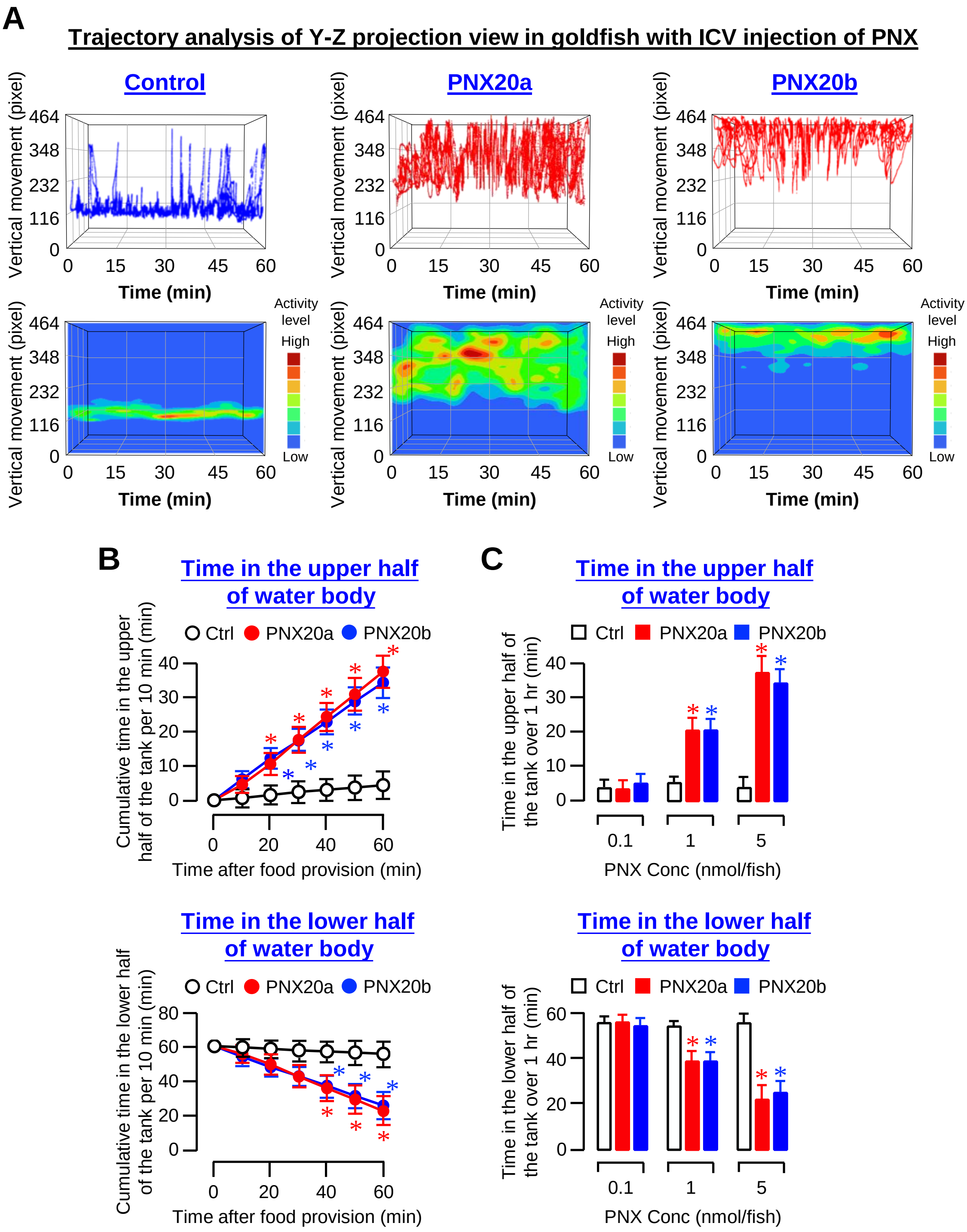

Supplementary Fig.8 Analysis of spatial preference of movement based on the Y-Z projection view of trajectory traces of goldfish with ICV injection of PNX20a/b. Vertical movement associated with feeding in goldfish with ICV injection (5 nmol/g BW) of PNX20a/b was recorded for the duration as indicated and with parallel injection of fish physiological saline as the control. The videos obtained were analysed with DeepLabCut and coordinate data for vertical movement with respect to time were extracted for construction of trajectory plots and heat maps for spatial preference of motility (A). For quantitative analysis of spatial preference, cumulative time for goldfish staying in the upper half (upper panels) and lower half of water body (lower panels) were calculated based on the trajectory traces for (B) time course study with ICV injection (5 nmol/g BW) of PNX20a/b up to 1 hr, and (C) dose-dependence study with ICV injection of increasing levels (1-5 nmol/g BW) of PNX20a/b (with drug treatment for 1 hr). An asterisk (*) represents a significant difference ($p < 0.05$) compared to the respective control.
